# Hepatic Spheroid Formation on Carbohydrate-Functionalized Supramolecular Hydrogels

**DOI:** 10.1021/acs.biomac.2c01390

**Published:** 2023-05-29

**Authors:** Jie Liu, Ying Zhang, Kim van Dongen, Chris Kennedy, Maaike J.G. Schotman, Patricia P. Marín San Román, Cornelis Storm, Patricia Y.W. Dankers, Rint P. Sijbesma

**Affiliations:** † Institute for Complex Molecular Systems, Department of Chemical Engineering and Chemistry, Eindhoven University of Technology, Eindhoven 5600 MB, The Netherlands; ‡ Institute for Complex Molecular Systems, Department of Biomedical Engineering, Eindhoven University of Technology, Eindhoven 5600 MB, The Netherlands; § CytoSMART Technologies B.V., Vrijstraat 9B, Eindhoven 5611 AT, The Netherlands; ∥ Institute for Complex Molecular Systems, Department of Applied Physics, Eindhoven University of Technology, Eindhoven 5600 MB, the Netherlands

## Abstract

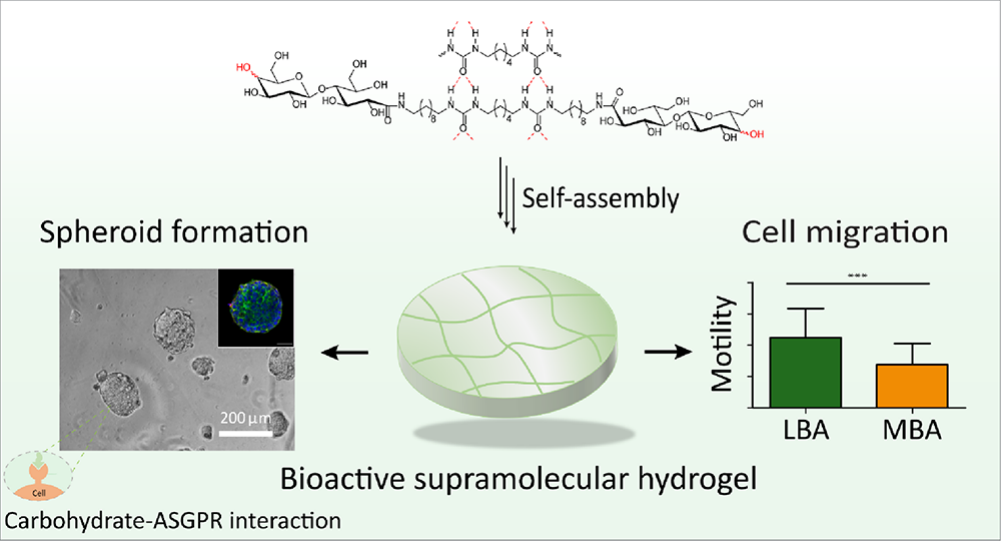

Two synthetic supramolecular hydrogels, formed from bis-urea amphiphiles containing lactobionic acid (LBA) and maltobionic acid (MBA) bioactive ligands, are applied as cell culture matrices *in vitro*. Their fibrillary and dynamic nature mimics essential features of the extracellular matrix (ECM). The carbohydrate amphiphiles self-assemble into long supramolecular fibers in water, and hydrogels are formed by physical entanglement of fibers through bundling. Gels of both amphiphiles exhibit good self-healing behavior, but remarkably different stiffnesses. They display excellent bioactive properties in hepatic cell cultures. Both carbohydrate ligands used are proposed to bind to asialoglycoprotein receptors (ASGPRs) in hepatic cells, thus inducing spheroid formation when seeding hepatic HepG2 cells on both supramolecular hydrogels. Ligand nature, ligand density, and hydrogel stiffness influence cell migration and spheroid size and number. The results illustrate the potential of self-assembled, carbohydrate-functionalized hydrogels as matrices for liver tissue engineering.

## Introduction

The extracellular matrix (ECM) is formed by hydrogel-like networks of fibrous proteins that provide cells with structural support and biochemical cues to direct cell growth and the phenotype.^1^ Although synthetic hydrogels offer precise control over composition and biophysical properties, commonly used hydrogels that are created by chemically cross-linked polymer networks fail to mimic the filamentous architecture and dynamic nature of extracellular matrices.^2^ Alternatively, physically cross-linked supramolecular hydrogels formed by hierarchical assembly of low-molecular-weight gelators (LMWGs) have emerged as an important subclass of biomimetic hydrogels, recapitulating the dynamic physical characteristics and fibrous microarchitectures of natural ECMs.

Urea,^3^ ureido-pyrimidinone,^4^ oligopeptide,^5^ and carbohydrate^6^ moieties have been incorporated into building blocks of LMMGs for the development of biomaterials. Among them, carbohydrate-containing LMWGs have received particular interest as they usually exhibit excellent biocompatibility, biodegradability, and promising mechanical properties.^6,7^ Carbohydrates contain multiple hydroxy groups and can provide robust inter- and intramolecular hydrogen bonding interactions, which have been extensively exploited to produce various dedicated and ordered nanostructures with a combination of other noncovalent interactions, such as hydrophobic and/or π-π interactions.^7−9^ Many examples have shown that carbohydrate-derived LMWGs show high potential for biomedical applications, including wound healing,^10^ cargo delivery,^11,12^ and cell culture.^9,13,14^ Despite this progress, most of carbohydrate based-LMWGs have not been examined for tissue engineering applications, and none has been shown to exhibit specific biological functions to promote cell-mediated remodeling.^7^ Additionally, a subtle change of carbohydrate configuration could lead to a dramatic difference in assembled morphologies and gelation ability,^15,16^ which significantly affects properties and potential applications of the resulting materials. Thus, judicious design of carbohydrate-based LMWGs is required to facilitate the formation of supramolecular hydrogels as ECM mimics.

Carbohydrates in biology serve as key elements in modulating cell adhesion, migration, differentiation, and regulation or controlling cellular behavior through cell surface receptors.^17,18^ For example, galactose (Gal) ligands are recognized by asialoglycoprotein receptors (ASGPRs) that are abundantly present on the surface of hepatocytes. The strong interaction between ASGPRs and Gal moieties can stimulate cell–matrix and cell–cell interactions and induce the formation of 3D hepatocyte spheroids.^19,20^ Such spheroids exhibit higher similarity to the biological tissue compared to monolayer cells, and their use has resulted in enhanced liver-specific functions for liver tissue engineering.^20,21^ Therefore, Gal-functionalized materials, in which biologically active Gal ligands are covalently grafted to polymers, have been a source of an artificial adhesion matrix for liver tissue engineering. Moreover, many researchers have found that glucose (Glc)-functionalized nanocarriers^22,23^ or glucose-based biopolymers^24−26^ can also be applied in hepatocyte-related biomedical engineering fields, including drug delivery. It has been demonstrated that, because of a lack of discrimination of ASGPR receptors between Gal and Glc configurations, the Glc moiety can bind to ASGPRs as well.^27,28^ However, the difference of the hepatic cell interaction with Gal- and Glc-based bioactive matrices remains unclear. Since supramolecular hydrogels have begun to emerge as attractive scaffolds for mimicking ECMs, the incorporation of Gal and Glc ligands in supramolecular monomers provides a promising platform to study hepatic cell behavior on two distinct bioactive matrices. It also opens an avenue to investigate the effect of the subtle structural variation of a single stereocenter on cell–cell and cell–matrix interactions. It has been reported that many biomimetic hydrogels have been used for the growth of the spheroids^29^ or organoids.^30,31^ For example, Kieltyka and co-workers recently reported a synthetic squaramide-based supramolecular hydrogel-functionalized with the Arg-Gly-Asp (RGD) motif. The gel showed bioactivity by supporting the formation of HepG2 spheroids over 3 weeks of culture.^32^

**Scheme 1:**
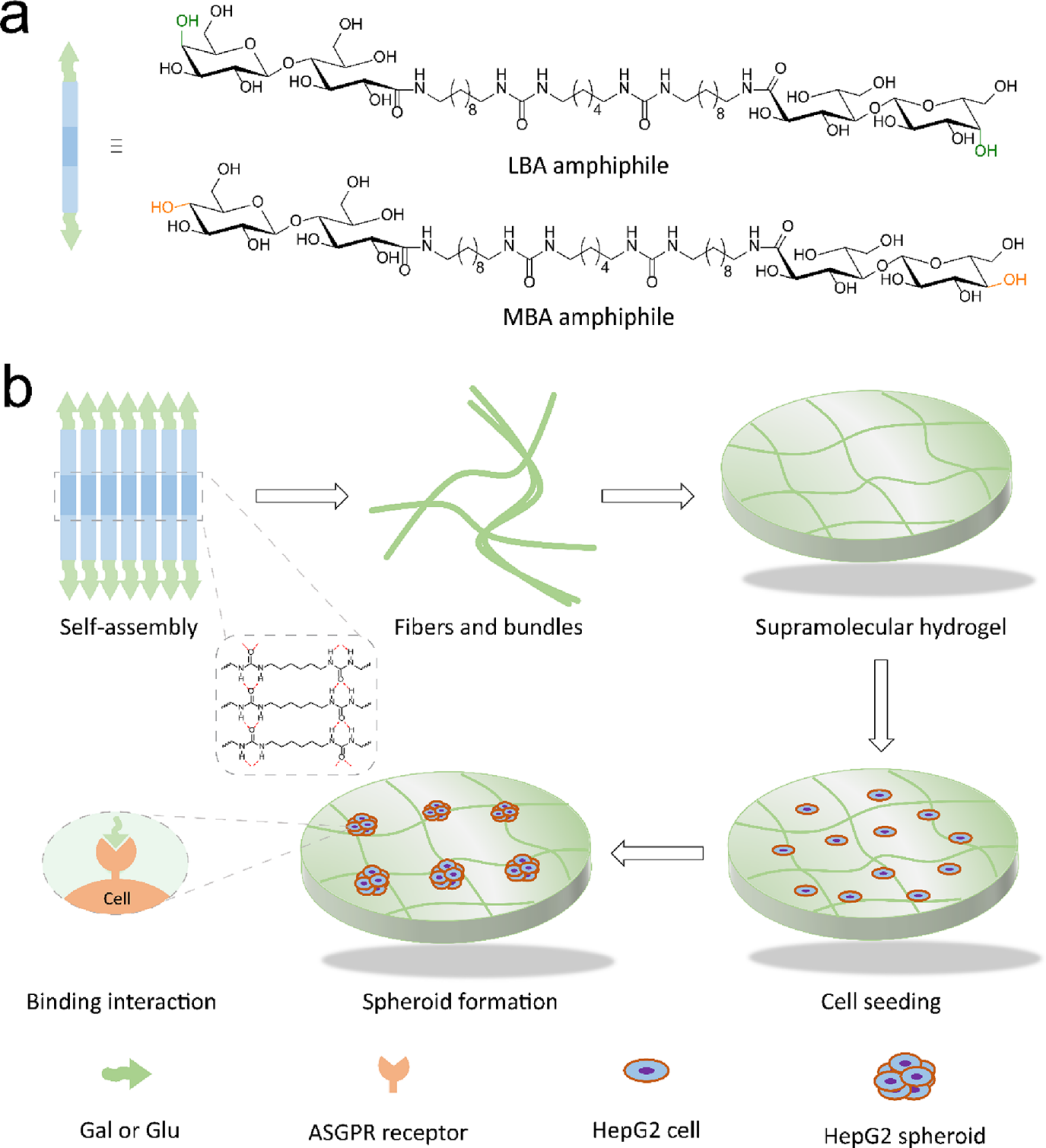
(a) Chemical Structures of Carbohydrate-Based Bis-urea Amphiphiles; (b) Schematic Illustration of Supramolecular Hydrogel Formation of Carbohydrate Functionalized Bis-urea Amphiphiles in Water and Cell Culture of HepG2 Cells on Bis-urea Amphiphile Hydrogels

Inspired by these considerations, we introduce isomeric lactobionic acid (LBA, Gal-based)- and maltobionic acid (MBA, Glc-based)-functionalized bis-urea amphiphiles, in which carbohydrate ligands are chemically conjugated to a hydrophobic alkyl core with two urea moieties (Scheme 1a). We first investigate how the self-assembly and gelation formation ability of carbohydrate amphiphiles in water are affected by carbohydrate stereochemistry. Subsequently, a commonly used human hepatic cancer cell line (HepG2) is exploited to elucidate how hepatic cells respond to different carbohydrate ligands on the gels and to explore the spheroid formation mechanism (Scheme 1b), tracked by time-lapse microscopy. Furthermore, the differences in spheroid formation on different gel substrates in terms of gel stiffness, ligand density, and binding interaction of different carbohydrate ligands are examined in a competition assay with two water-soluble MBA- and LBA-based inhibitors.

## Experimental Section

### Synthesis of Carbohydrate-Based Bis-urea Amphiphiles

Detailed synthesis procedures are described in the Supporting Information.

### Preparation of Carbohydrate-Based Amphiphile Solutions and Gels

A carbohydrate-based amphiphile was dissolved in Mili-Q water with sonication to get an opaque suspension, which was heated in a sealed vial in an oil bath for 10 min at 120 °C and then cooled quickly in an ice bath to obtain a clear solution or gel. The solutions or gels were allowed to reassemble for at least 1 day. Samples were diluted with Mili-Q water prior to imaging.

### Critical Micellization Concentration (CMC) Measurements

Nile red is a hydrophobic and solvatochromic dye that is commonly used to determine CMC values of supramolecular assemblies. A series of 1.0 mL of carbohydrate amphiphile solutions were prepared by diluting 8.4 mM (10 mg/mL) stock solution with Mili-Q water. Next, the solutions were heated in a water bath at 100 °C for 5 min, quickly quenched in ice water, and equilibrated by standing at room temperature for 3 days before adding Nile red solution. Finally, 5.0 μL of 0.2 mM Nile red in DMSO was added into 1.0 mL of carbohydrate amphiphile solutions, and all samples were equilibrated for at least 2 h in the dark before measuring. A Perkin Elmer Luminescence Spectrometer LS 45 was used to measure CMCs for samples. The fluorescence of Nile red was recorded from 560 to 800 nm with a voltage of 800 V and a slit of 5 nm, with an excitation wavelength of 550 nm. The ratio of intensity at 636 nm (the emission maximum of the dye in the hydrophobic environment) to that 655 nm (the emission maximum in aqueous conditions) was then plotted against the concentration of each carbohydrate amphiphile.

### Cryogenic Transmission Electron Microscopy (Cryo-TEM)

Samples for cryogenic transmission electron microscopy were prepared in an automated vitrification robot (VitrobotTM Mark IV, FEI company) at room temperature and a relative humidity >95%. Samples (3 μL) were applied on Quantifoil grids (carbon support film on a copper grid, type R 2/2, Electron Microscopy Sciences) or Lacey grids (LC200-Cu, Electron Microscopy Sciences), which were glow discharged prior to use (Cressington 208 carbon coater operation at 5 mA for 40s). Subsequently, excess liquid was blotted away and vitrified in liquid ethane. The vitrified grids were examined on a FEI-TITAN TEM equipped with a field emission gun operating at 300 kV. Samples were imaged using a post-column Gatan energy filter and 2048 × 2048 Gatan CCD camera. Vitrified films were examined at temperatures below −170 °C at low-dose conditions. Magnifications of 6500 with a defocus setting of −40 μm, and 24,000 with a defocus setting of −10 or −5 μm were used. ImageJ software was used for image analysis.

### Atomic Force Microscopy (AFM)

Atomic force microscopy was carried out on a Digital Instruments Dimension Nanoscope IV in tapping mode regime to record height images with silicon cantilever tips (PPP-NCHR, NanoSensors, 204–497 kHz, and 10–130 N·M^–1^) at room temperature. Diluted samples (10 μL) were placed on a VI Mica disc (12 mm, Ted Pella Inc.) for 10 min and then were washed with 200 μL of Mili-Q water three times using pipette tips. Subsequently, the samples were dried in an N_2_ flow atmosphere overnight before measurement. Images were processed by using NanoScope Analysis Software (version 1.9).

### Small-Angle X-Ray Scattering (SAXS)

Small-angle X-ray scattering (SAXS) profiles were recorded on a SAXLAB GANESHA 300 XL SAXS equipped with a GeniX 3D Cu Ultra-Low Divergence microfocus-sealed tube source producing X-rays with a wavelength λ = 1.54 Å at a flux of 1 × 10^8^ ph/s and a Pilatus 300 K silicon pixel detector with 487 × 619 pixels of 172 × 172 μm^2^ in size placed a three sample-to-detector distance of 113, 713, and 1513 nm, respectively, to cover a *q*-range of 0.1 ≤ *q* ≤ 4.0 nm^–1^ with *q* = 4π / λ · (sin θ / 2). The two-dimensional images were averaged to obtain the intensity *I*(*q*) vs *q* profiles and calibrated to absolute scale using Mili-Q water as a reference, standard data reduction procedures, i.e., subtraction of the empty capillary and the solvent contribution, were applied. The samples were prepared at a concentration of 10 mg/mL in Mili-Q water and held in 2 mm quartz capillaries. Small-angle X-ray scattering experiments were performed at 20 °C.

### Rheological Measurements

The mechanical properties of the hydrogels were performed on a Physicia MCR 501 Discovery HR-3 oscillatory rheometer, equipped with a 25 mm stainless steel sand-blasted plate-plate geometry to prevent sample slippage. The sample volume was 300 μL at a fixed plate-to-plate gap of 500 μm, and mineral oil was placed around the sample to minimize evaporation. The temperature was fixed to 37 °C, and gelation was monitored under an oscillatory strain of 1.0% and an angular frequency of 1.0 Hz. The frequency sweep was conducted under a fixed amplitude of 1.0%, followed by a strain sweep with a fixed angular frequency of 1.0 Hz. The self-healing behavior was measured continuously at a fixed angular frequency of 1.0 Hz, and the breakage of the gel network was at a maximal strain of 500.0%, and the recovery stain was at a minimal strain of 1.0%.

### Cell Culture Experiments

HepG2 cells were cultured in a DMEM medium containing 10% FBS, 1% penicillin/streptomycin (complete DMEM) in 5% CO_2_ at 37 °C.

### Gel Preparation for the Cell Culture

The gels stored at 4 °C were taken out of the fridge and briefly shaken for seconds to obtain solutions, and the resulting solutions were pipetted into μ-Slide angiogenesis plates for gelation. The solutions were allowed to reassemble for 1 day at room temperature. The recovered gels and plates were treated with UV light for 10 min to sterilize before cell seeding. Subsequently, the cells were seeded on the top of hydrogels at a density of 2000 cells per well and cultured at 37 °C for 3–5 days. The optical images of cells morphology were recorded on optical microscopy equipped with a Leica camera.

### Live/Dead Staining

For the live/dead cell staining, the HepG2 cells were washed with PBS twice, loaded with 1 μmol calcein and 2 μmol propidium iodide (PI) for 1 h, and then directly captured using a Leica SP8 system. Empty wells with a complete DMEM medium were used as the control group, and all experiments were conducted in triplicate.

### Urea and Albumin Assays

Three batches of cells of different conditions were cultured at the same time to harvest the medium on day 3, day 5, and day 7. When the cells were ready, the medium were collected and frozen at −20 degree for later analysis. After all the samples were collected, they were analyzed based on the instructions from the manufacturers. The urea kit was purchased from BioAssay Systems (QuantiChromTM Urea Assay Kit (DIUR-100)), and the albumin assay kit was from Thermo Fisher (The Human Albumin (ALB) ELISA Kit).

### Immunofluorescence Staining

For 2D-cultured HepG2 cells immunofluorescence analysis, spheroids generated on gels were fixed with 3.7% formalin for 30 min, followed by further washes with PBS. The cells were permeabilized with 0.2% Triton X-100 in PBS for 1 h, followed by another 1 h of blocking with 2% BSA in PBS. The primary antibody incubation was performed in 1% BSA in PBS at room temperature for 2 h. The following primary antibodies were used: mouse β1-integrin (Santa Cruz, Cat. #sc-53711, 1:200) and rabbit Ki-67 (Thermo scientific, #rb1510-P0, 1:500). The secondary antibody incubation was in 1% BSA in PBS at room temperature for 1 h, followed by three PBS washes. Alexa 488 (Invitrogen, Cat. #A21206, 1:400)- or 555 (Invitrogen, Cat. #A21424, 1:400)-conjugated secondary antibodies were used. All immunofluorescence experiments were performed with negative controls without any primary antibodies. The samples were then incubated with DAPI (5 μg/mL) at room temperature for 10 min, followed by washing by PBS three times. The samples were mounted in PBS. Alexa-647-conjugated phalloidin (Life Technologies) was used 1:100 in 1% BSA to visualize F-actin microfilaments. Images were acquired using a Leica TCS SP8X confocal microscope. Acquired images were processed with Fiji, a newer processing package based on ImageJ.

### Time-Lapse Microscopy

Time-lapse microscopy was conducted to analyze cell motility, migration behavior, morphology, and spheroid formation. The supporting videos were recorded on a CytoSMART Lux3 BR, placed in an incubation system. Each gel was imaged using one field of view for a period of up to 5 days, with an interval of 5 min. A Leica DMi8 microscope was used for trajectory analysis with an interval of 3 min. The images were analyzed using ImageJ software. To determine cell trajectories, migration paths of 20–30 random cells under each condition (*n* = 3) were manually tracked using the ImageJ plugin MTrackJ (Erasmus University Medical Center Rotterdam, the Netherlands). The trajectories were analyzed for 10 h after seeding. The migration paths were established and analyzed in ImageJ with Chemotaxis and Migration Tool (version 2.0, ibidi GmbH, Germany). To quantify cell velocity, cells were automatically tracked using the ImageJ plugin TrackMate (version 4.0.0) for 10 h (*n* > 20 for each condition). To reduce plate drift, image stacks were preprocessed with the plugin “Manual drift correction”. The image stacks were then loaded into TrackMate, which detects the cell locations using the “Simple LAP tracker algorithm” and generates cell trajectory paths. For each validated track, the velocity and track duration are automatically calculated.

### Competition Assay

Different concentrations of GalNAc, Butyl-LBA, and Butyl-MBA (50, 100, and 300 mM, respectively) were mixed with a cell suspension on 17 mM LBA gel. After 48 h culture, the resulting cell clusters were recorded by bright-field imaging and analyzed with Fiji.

## Results and Discussion

### Synthesis and Self-Assembly Behavior

Carbohydrate-based amphiphiles were synthesized by a facile sequence of synthetic steps in direct analogy to the synthesis of a nonbioactive oligo(ethylene glycol) (OEG) amphiphile as previously reported.^33^ Synthesis started with the acid-catalyzed ring-closure reaction of bionic acid, which significantly increases the reaction rate with diamines,^34^ followed by adding a 3-fold excess of decyl diamine to minimize the formation of symmetrical side product with two carbohydrate groups. In the last step, 0.5 equiv of hexamethylene diisocyanate was added to obtain the final product (Schemes S1 and S2). A combination of precipitation and recrystallization provided the product with a satisfactory purity (>95%, determined by ^1^H NMR) and yield (∼65%). The details of the synthesis and chemical characterizations of two carbohydrate amphiphiles are presented in the Supporting Information, Figures S1–S6.

LBA and MBA are water-soluble stereoisomers, thus serving as head groups to increase water solubility and to balance the hydrophobic/hydrophilic ratio of bis-urea amphiphiles. The bis-urea amphiphiles self-assemble in water, driven by the hydrophobic interaction, which is strengthened and rendered more directional by hydrogen bonding of the urea groups (Scheme 1b).^35^ It is well-known that a subtle change in the molecular structure of supramolecular monomers often results in markedly different assembled morphologies and gelation behavior.^36,37^ Therefore, the critical micelle concentration (CMC) of the two carbohydrate amphiphiles was first investigated using the Nile red assay. Both amphiphiles exhibit a low CMC, with the LBA amphiphile (2.3 μM) having a slightly lower CMC than the MBA amphiphile (4.8 μM), as indicated in Figure S9.

**Figure 1:**
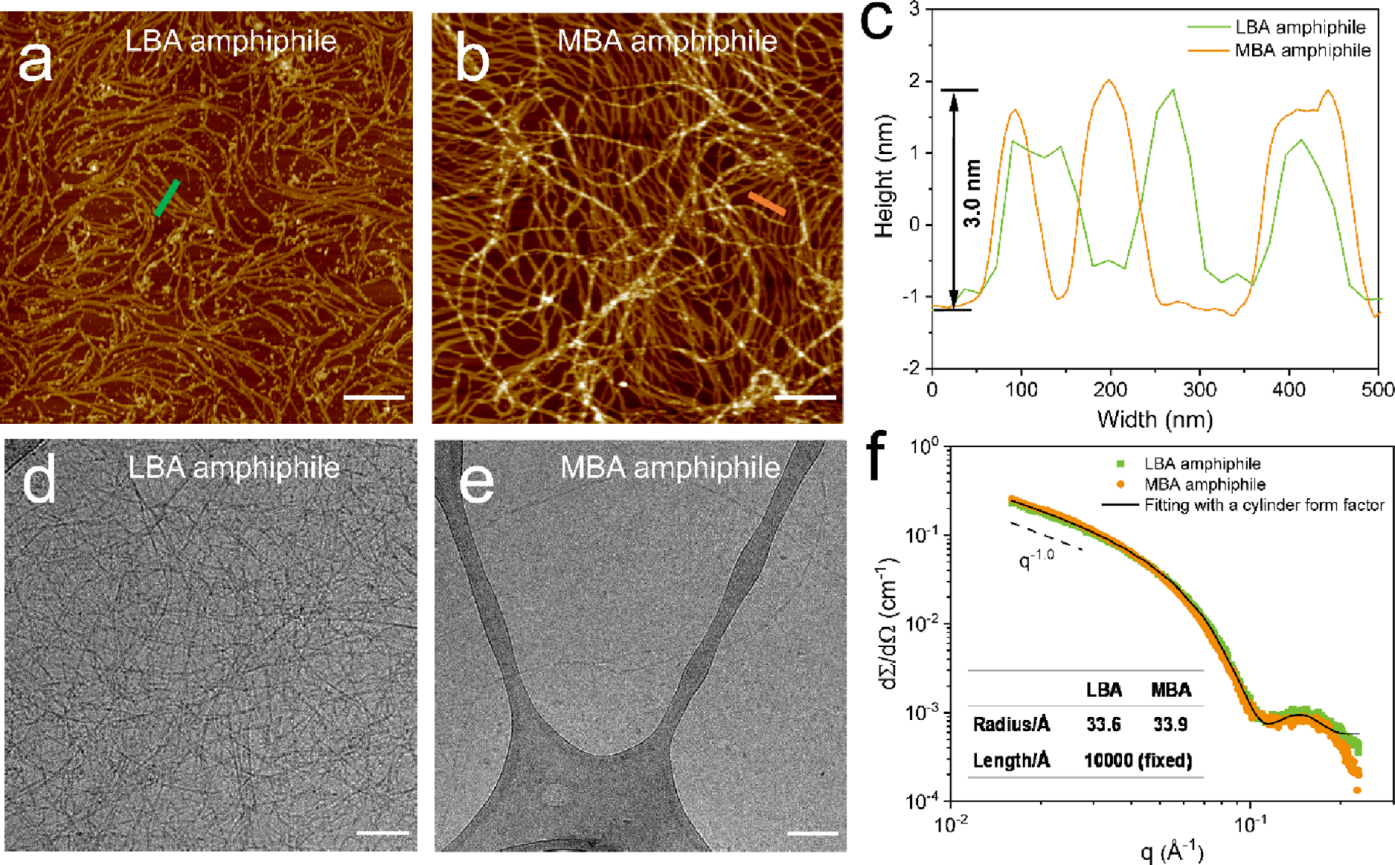
Self-assembled morphology of carbohydrate-based bis-urea amphiphiles in water. Representative AFM images of the LBA amphiphile (a) and the MBA amphiphile (b) at 0.1 mM on fresh mica by a drop-casting method. The scale bar is 1.0 μm. (c) Height profile of LBA and MBA amphiphile fibers, indicated by thick-colored lines. Representative Cryo-TEM images of LBA amphiphile (d) and MBA amphiphile (e) at 0.5 mM. The scale bar is 100 nm. (f) SAXS profiles (symbols) and form factor fits (lines) for carbohydrate amphiphiles in water at a concentration of 8.4 mM (10 mg/mL). Insert table: fitting results based on a cylindric form factor model.

The morphology of the aggregates of the two amphiphiles in solution (above CMC) was further examined with AFM. To this end, diluted solutions were heated to 120 °C in sealed vials to erase thermal history. Upon quenching in an ice bath, both LBA and MBA amphiphiles assemble into fibers with a length of several micrometers (Figure 1a,b and Figure S10) with a uniform height of ∼3.0 nm and a cross-sectional width of ∼100 nm (Figure 1c) observed in AFM images. The large observed fiber cross-section in both amphiphiles is likely related to the synergistic result of fiber aggregation during drying and convolution of fiber width with the radius of the tip.^38^ The assembled morphologies were also visualized by cryo-TEM, and both amphiphiles form well-defined fibers with a length of several micrometers and a uniform diameter of 5–6 nm (Figure 1d,e and Figure S11), but fewer fibers were observed for the MBA amphiphile at the same concentration (0.5 mM) after aging 48 h (Figure S12). It is also notable that, at a lower concentration (0.1 mM), numerous long fibers were still observed for LBA amphiphiles but not for MBA amphiphiles (Figure S13). These results prove that the LBA amphiphile assembles into long fibrous aggregates at a lower concentration, in good agreement with the lower CMC value of the LBA amphiphile. As compared to short rodlike micelles in the nonbioactive analogue OEG amphiphile, the formation of super long fibers in carbohydrate amphiphiles can be explained by the presence of multiple hydrogen bonds between carbohydrates, making them stack easily along the direction of fiber growth. It has been reported that carbohydrate-mediated multivalent interactions are important to form stable, discrete nanostructures stabilized by inter- and intralayer lateral hydrogen bonding between carbohydrate head groups.^39−41^

The fiber morphology of the two amphiphiles was also investigated with small-angle light scattering technique (SAXS), and notably, the two scattering profiles of 8.4 mM (10 mg/mL) aqueous solutions overlapped in the whole *q* regime (Figure 1f), revealing that LBA and MBA amphiphiles assemble into nanostructures with a very similar molecular packing. The shape of the aggregates can be directly derived from ∼ the slope of the SAXS profiles, and both carbohydrate amphiphiles exhibit a *I* ∝ *q*^–1.0^ power-law regime, characteristic of rod-like objects. At high *q* values, near 0.15 Å^–1^, a striking scattering peak was observed in both carbohydrate amphiphiles, corresponding to the diameter of fibers. To further quantify the dimensions of the aggregates, the scattering profiles were fitted assuming a homogenous flexible cylindrical form factor. The profiles of two carbohydrate amphiphiles are well-fitted as objects with a simple cylindrical form factor, providing a radius of 3.4 nm. Unfortunately, it was not possible to extract reliable fiber lengths from the scattering profiles because this parameter is far beyond the experimental resolution of the instrument. Therefore, the length parameter was fixed at 1.0 μm during fitting considering that the very long fibers of two amphiphiles were observed in the microscopy images. The fitted radii for the carbohydrate amphiphiles are close to the fiber radius of 3.5 nm for the previously reported OEG amphiphile^33,42^ and are also consistent with the diameters visualized by cryo-TEM images (Figure 1d,e).

### Hydrogel Formation and Rheological Characterization

**Figure 2:**
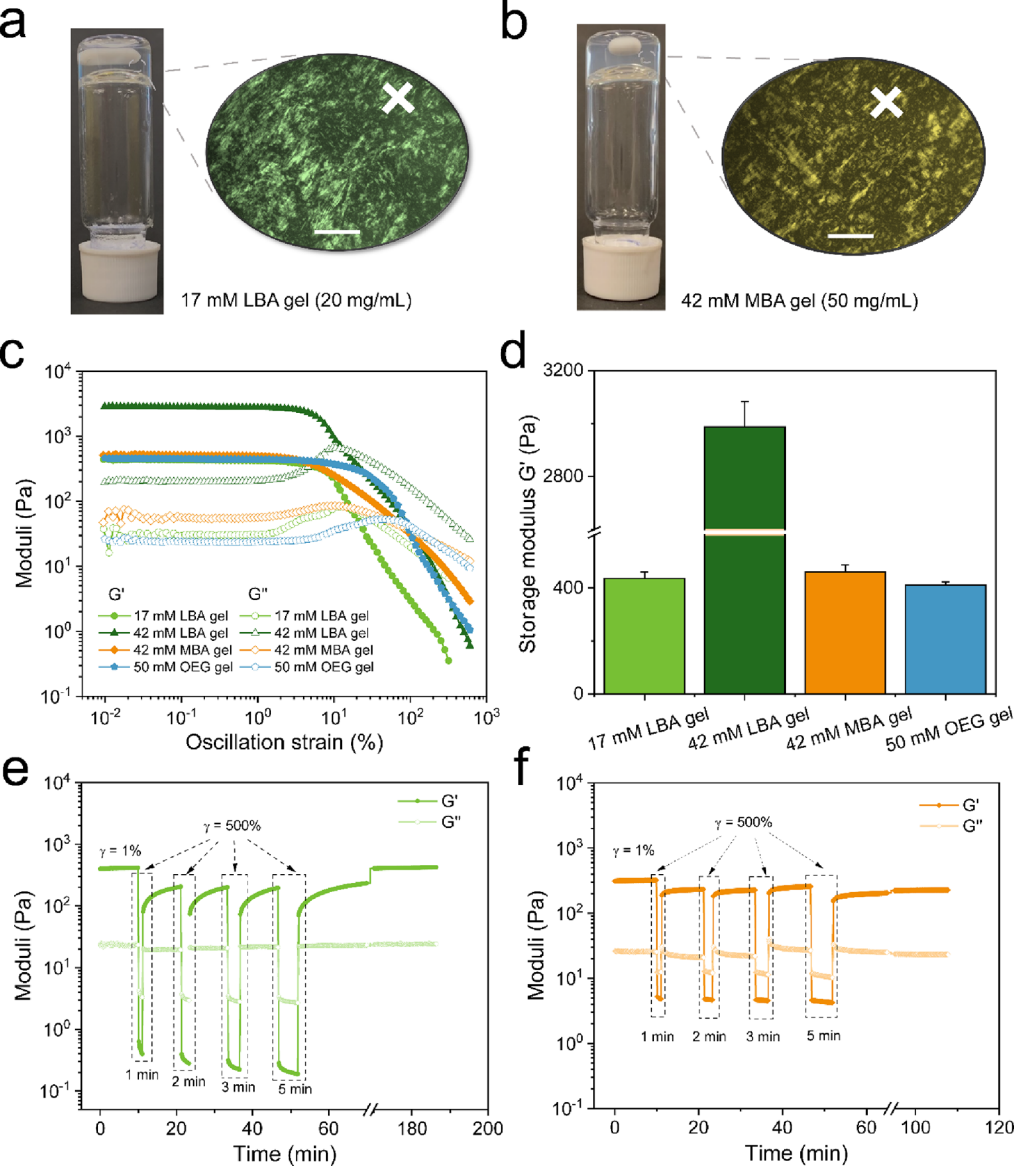
Gelation and rheological measurements of carbohydrate-based bis-urea amphiphiles in water. Optical and polarized light images of 17 mM LBA gel (a) and 42 mM MBA gel (b). The scale bar in POM images is 500 μm. (c) Amplitude sweeps of different amphiphile hydrogels (storage modulus G′ and loss modulus G″). (d) Summary of G′ for different amphiphilic gels. Self-healing properties of 17 mM LBA (e) and 42 mM MBA (f) hydrogels at a minimum strain of 1.0% (recovery) and a maximum strain of 500.0% (breaking) with an interval of 1, 2, 3, and 5 min, respectively. The measurement temperature was set up at 37 °C.

As visualized in microscopy images, the long fibers in LBA and MBA amphiphiles tended to be twisted together and form network-like structures, suggesting that they can form gels by aggregating into a supramolecular polymer network at higher concentration. Upon heating, the carbohydrate amphiphiles dissolved readily in water, and rapidly cooling, the aqueous solutions in an ice bath gave transparent hydrogels upon standing at room temperature, indicating that both amphiphiles are LMWGs. However, slow cooling of the solutions resulted in opaque and inhomogeneous suspensions or gels (Figure S14), possibly due to the formation of large crystalline structures, strongly implying crystallization-driven self-assembly. Surprisingly, under polarized light, both amphiphilic gels were birefringent (Figure 2a,b and Figure S15), a characteristic of lyotropic liquid crystal phases of glycolipids systems in which tight stacking of carbohydrate amphiphile molecules results in the formation of large fiber bundles.^43,44^ Such bundles were observed from cryo-TEM images of 17 mM LBA gel, as indicated in Figure S16.

Rheological testing of LBA, MBA, and OEG amphiphiles was performed with an oscillatory rheometer, and all hydrogels consistently displayed a viscoelastic response to oscillatory shear, with a storage modulus (G′) that exceeded the loss modulus (G″) by an order of magnitude (Figure 2c). The LBA amphiphile formed a stable hydrogel more quickly than MBA and OEG amphiphiles. For example, a 17 mM solution of LBA amphiphile gel reached a plateau modulus within ∼3 h, while 42 mM MBA and 50 mM OEG hydrogels required ∼10 h (Figure S17a). The gel concentrations required to reach a similar G′ of around 400 Pa for LBA and MBA amphiphiles were 17 and 42 mM, respectively, while the OEG amphiphile required the highest gel concentration (50 mM) upon heating–cooling (Figure 2c,d). The OEG amphiphile with the same hydrophobic core and bis-urea moiety only forms short fibers in water,^33^ thereby suggesting that the two carbohydrate amphiphiles (LBA and MBA) aggregate through intermolecular hydrogen bonds not only between bis-urea groups but also between alcohol residues of carbohydrate moieties.

An LBA amphiphile gel at 42 mM was also prepared, and it had a G′ of ∼2900 Pa, which is 6 times higher than the MBA amphiphile at the same concentration (Figure 2d). The difference in the storage modulus is likely related to the formation of three-dimensional hydrogen-bond networks by the galactose moieties in the LBA amphiphile, while the glucose in the MBA amphiphile only form two-dimensional networks, leading to a weaker gel with less intermolecular hydrogen bonding interactions.^40^ The formation of gels from these carbohydrate amphiphiles is therefore probably not just the result of fiber entanglement but is also caused by bundle formation. As indicated in Figure 2e,f, both carbohydrate gels self-heal after a high strain-induced breaking (γ = 500%), followed by a gradual full recovery at a low strain (γ = 1.0%), confirming the dynamic nature of these supramolecular gels, and giving promising applications in injectable scaffolds for tissue engineering.

### Spheroid Formation on Carbohydrate Hydrogels

**Figure 3:**
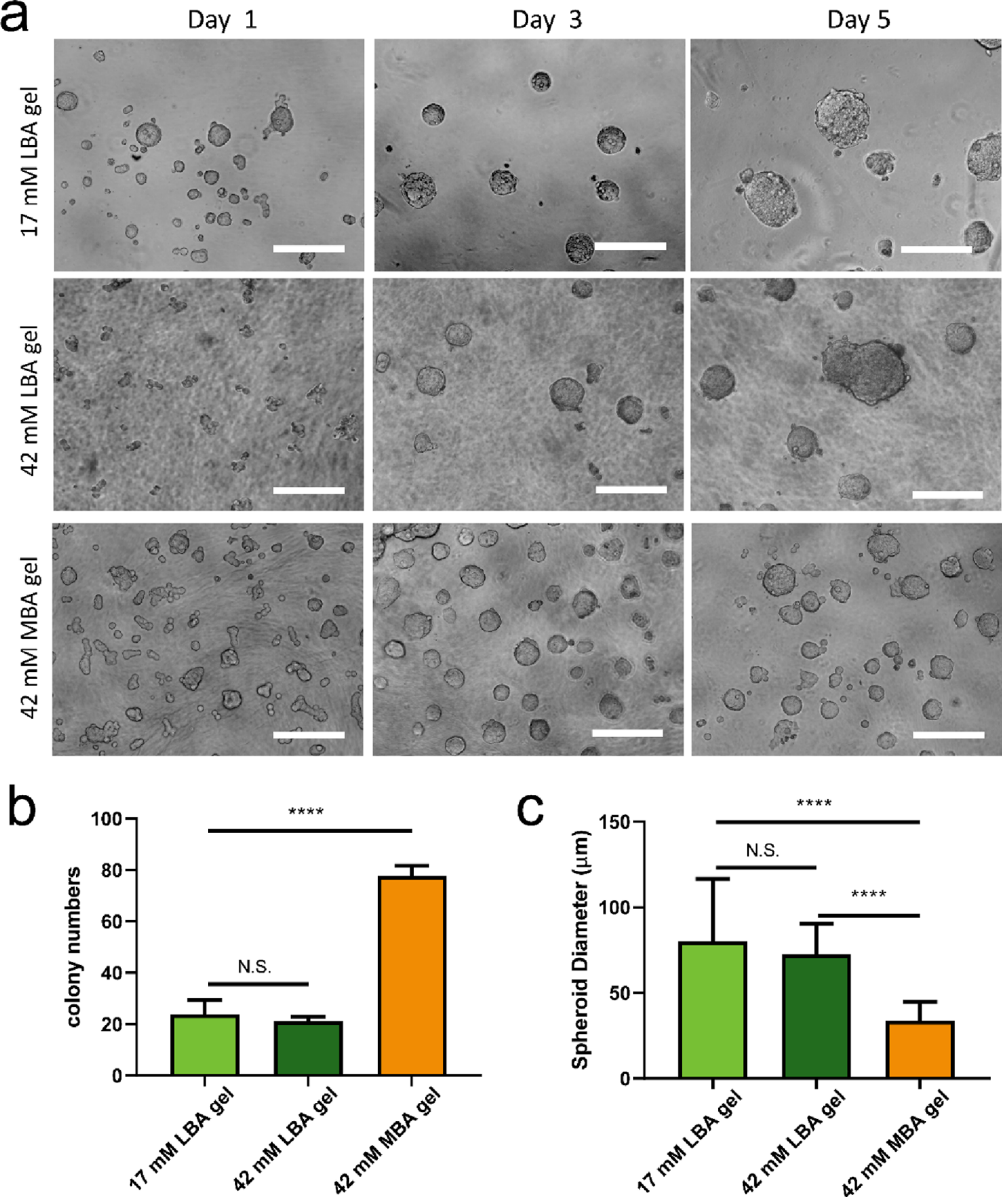
Spheroid formation on carbohydrate amphiphilic gels. (a) Optical contrast microscopic observation of HepG2 cell behavior on 17 mM LBA, 42 mM LBA, and 42 mM MBA amphiphile gels over a period of 5 days after seeding. The scale bar is 200 μm. Statistical analysis of spheroid colony (b) and diameter (c) at day 5 on LBA and MBA amphiphile gels. Quantitative measurement of spheroid size and diameter was conducted by image processing analysis software (ImageJ). Statistically significant differences on spheroid colony and diameter were assessed by using one-way ANOVA followed by Tukey’s multiple comparison test. *P* values of statistical significance are represented as * *P* < 0.05, ** *P* < 0.01, *** *P* < 0.001, N.S., not significant.

Carbohydrate ligands in synthetic substrates are known to bind to specific ASGPR receptors presented at the cell surface of hepatocytes. These specific cell-ECM interactions induce the formation of spheroids with improved liver function compared to traditional 2D monolayers.^45,46^ To investigate the effect of different ligand categories and carbohydrate ligand density in the self-assembled gels on spheroid formation, 17 mM LBA and 42 mM MBA gels were prepared. Note that both have a storage modulus (G′) of ∼400 Pa, similar to that of the stiffness of a normal liver *in vivo*,^47^ and the self-healing properties of both gels indicate that they are sufficiently dynamic to mechanically adjust to cells. Next to this, a 42 mM LBA gel was prepared with the same carbohydrate ligand density as the 42 mM MBA gel, but with a higher G′ of ∼2900 Pa. Phase-contrast microscopy images of HepG2 cultured on each matrix were recorded at day 1, day 3, and day 5. Notably, both carbohydrate amphiphile gels supported the formation of three-dimensional aggregates (so-called spheroids) within 24 h from HepG2 cell seeding (Figure 3a, Figures S18 and S19). However, the spheroids on the different substrates showed large differences in average diameter and number of colonies. Quantitative measurement of spheroids cultured for 5 days was conducted by image processing analysis software. Identification of 24 spheroids on a randomly selected area of 17 mM LBA gel provides an average diameter of 80 μm. No significant differences in average spheroid diameter and colony numbers were measured on LBA gels at two different concentrations (17 mM and 42 mM LBA) with G′ values of 400 and 2900 Pa, respectively. However, more spheroids (78) with a smaller size (diameter: 34 μm) were observed when culturing on 42 mM MBA gel (Figure 3b–c).

The OEG gel and collagen-coated well-plates were also investigated as controls. Interestingly, a large majority of cells remained dispersed on the nonadhesive OEG gel, and a spread-out morphology with monolayers was observed on collagen-coated plates over time (Figure S20). Altogether, these results show that both LBA and MBA gels promote the formation of the spheroids, but their differences in concentration, related stiffness, and ligand density may result in variations of spheroid diameters and formation efficiency.

**Figure 4:**
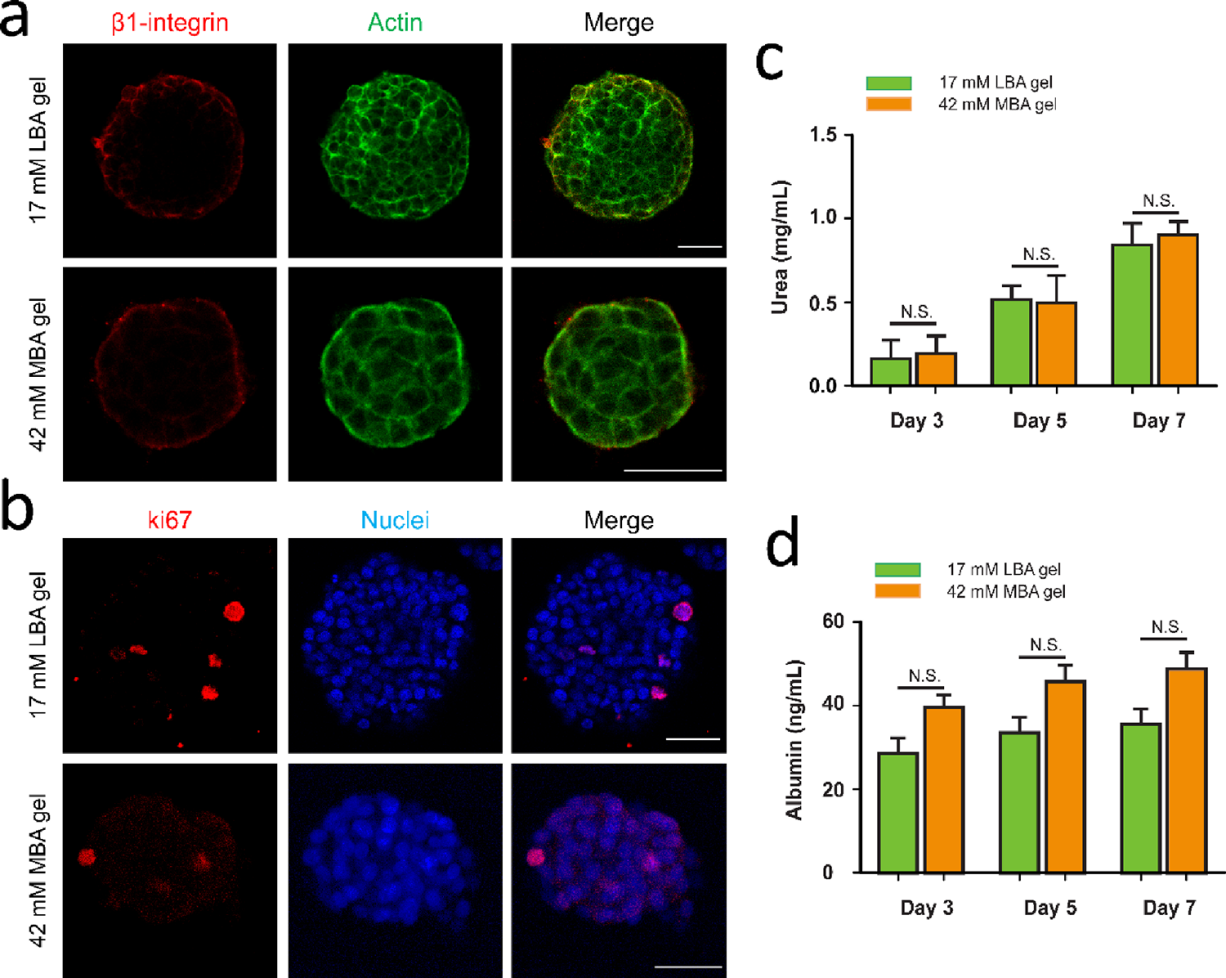
Spheroid staining and liver-specific function of spheroids. (a) Confocal microscopy of stained spheroids on 17 mM LBA gel and 42 mM MBA gel at day 5: (a) red: β1-integrin, green: actin, and merged images; (b) red: Ki-67, blue: nucleus and merged images. The scale bar is 50 μm. Urea (c) and albumin (d) production of HepG2 spheroids on LBA and MBA gels.

Importantly, the HepG2 cells in spheroids remained viable without observation of necrotic core on 17 mM LBA, 42 mM MBA, and 42 mM LBA gels after 5 days of culturing, suggesting low toxicity of these hydrogels to HepG2 cells (Figure S21). In spheroids with a size below 200 μm, diffusion limitations in the transportation of nutrients, oxygen, and waste are minimal and the formation of a necrotic core was not observed.^48,49^ Additionally, immunofluorescence staining experiments on F-actin and β1 integrin were performed as these are representative elements to demonstrate the structure of the spheroids, whose formation relies on the cell–matrix interaction. Intense staining of an intricate network of cytoskeletal F-actin (green) filaments was seen throughout the spheroids. Immunostainings for β1 integrin were mainly visualized at the margins of the spheroids where cells contact the gel matrix after culturing for 5 days (Figure 4a and Figure S22). From the above results, it is concluded that HepG2 cells remain viable as spheroids and display interactions with carbohydrate-functionalized hydrogels.

Cell proliferation of spheroids was also investigated by using Ki-67, a proliferation marker. The expression of Ki-67 was significantly low at day 3, 5, and 7, indicating that cell proliferation was low during the spheroid formation process (Figure 4b, Figures S23 and S24), in line with an observation from Price and co-workers.^50^ Moreover, the metabolic activity of spheroids generated on two different gels was investigated by evaluating albumin secretion and urea synthesis, two typical functions of liver hepatocytes at day 3, day 5, and day 7. Spheroids generated on 17 mM LBA and 42 mM MBA gels produced the same amount of urea and albumin, although the spheroid sizes and numbers on the two gels are significantly different, as indicated in Figure 4c,d. In liver spheroids, it has been reported that the metabolic and synthetic genes are upregulated compared to monolayer cells, thus leading to enhanced liver-specific functions,^51^ while the spheroid formation has been reported to inhibit the proliferation of cells via contact inhibition.^52^

### Inhibition of Spheroid Formation

**Figure 5:**
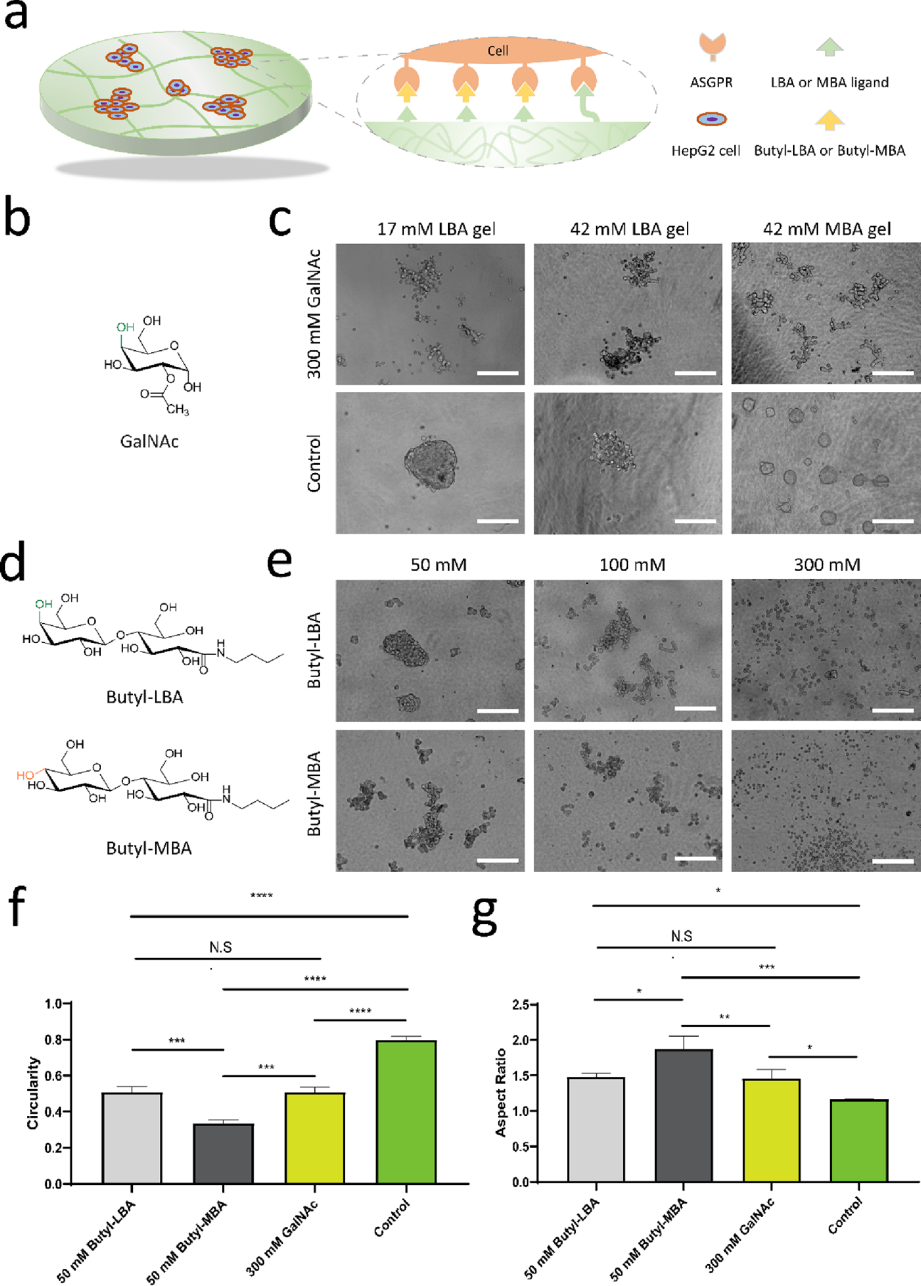
Competition assay with different analogous inhibitors. (a) Schematic illustration of binding competition between carbohydrate ligands in the gel matrix and carbohydrate ligands in inhibitors. (b) Chemical structure of the commercialized ASGPR inhibitor-GalNAc. (c) Representative bright-field images of HepG2 cells treated with 300 mM GalNAc and cells treated with PBS as the control group after 2 days of culturing on different gels. The scale bar is 200 μm. (d) Chemical structures of two inhibitors: Butyl-LBA and Butyl-MBA. (e) Representative bright-field images of cells treated with Butyl-LBA and Butyl-MBA (50, 100, and 300 mM), respectively. The scale bar is 200 μm. (f) Quantitative analysis of circularity, comparing the perimeter of a shape to the area it contains, of cell spheroids or clusters grown on a 17 mM LBA hydrogel. (g) Quantitative analysis of the aspect ratio, defined as the height over width dimension, of cell spheroids or clusters grown on a 17 mM LBA hydrogel. For each group, 10–20 spheroids or clusters were analyzed. The statistical significance on shape-related characteristics of spheroids was determined using unpaired two-sided *t*-test. *P* values of statistical significance are represented as * *P* < 0.05, ** *P* < 0.01, *** *P* < 0.001, N.S., not significant.

A competitive assay is an indirect measurement to determine specific interaction between ligands and protein receptors,^53^ and the schematic diagram of such competition with different analogous carbohydrate molecules is illustrated in Figure 5a. To investigate that spheroid formation is mediated through specific recognition between ASGPRs and carbohydrate ligands, *N*-acetylgalactosamine (GalNAc), a Gal-derived inhibitor that can bind to ASGPRs in the HepG2 cell membrane,^54^ was first used as a competitive ligand in the carbohydrate amphiphile gel system (Figure 5b). To this end, the different concentrations of GalNAc solutions (50, 100, and 300 mM) were mixed with the cell suspensions before seeding cells on the gels. The HepG2 cells still form compact aggregates in the presence of 50 and 100 mM GalNAc after 2 day culturing. Interestingly, cells treated with 300 mM GalNAc clustered into irregular and branched morphologies. That was in sharp contrast with the round and compact shape of spheroids observed in reference experiments in which the cells were treated with PBS (Figure 5c and Figure S25). These indicate that, to suppress the spheroid formation, the concentration of the GalNAc inhibitor present in the medium should be much higher than that of the carbohydrate ligands in the gels. Inhibition of spheroid formation by GalNAc also implies that the induction of spheroid formation by the LBA or MBA ligands in the gels is indeed mediated through ASGPRs.

It is thus clear that spheroid formation on LBA and MBA gels is mediated by ASGPRs-carbohydrate interactions. However, it remains unclear whether the configurational difference between LBA and MBA amphiphiles affects binding interaction to ASGPRs in HepG2 cells. To answer this question, two water-soluble compounds Butyl-LBA and Butyl-MBA were synthesized as analogous inhibitors to probe the difference in interactions to the ASGPR receptors (Figure 5d).^26,55^ Competition experiments were carried out on 17 mM LBA gels, to which different concentrations (50, 100, and 300 mM) of Butyl-LBA and Butyl-MBA were added by mixing with cell suspensions before cell seeding, followed by observation of aggregate morphology by bright-field microscopy, as shown in Figure 5e.

To determine the inhibitory effect of the two inhibitors, shape-related characteristics including circularity and the aspect ratio were quantitatively analyzed for these irregular cell aggregates with image analysis software. As shown in Figure 5f,g, compared to the control substrate (17 mM LBA gel without treatment), the circularity of all cell aggregates formed in the presence of one of these inhibitors became significantly smaller, and the aspect ratio increased after 2 days of culturing, suggesting the inhibition effect of all the reagents. The HepG2 cells still formed compact aggregates with 50 mM Butyl-LBA, leading to the circularity of 0.51 and an aspect ratio of 1.48, whereas the aggregates formed in the presence of 50 mM Butyl-MBA exhibited loose morphologies with the lower circularity and higher aspect ratio. These results suggest that Butyl-MBA in the medium has a stronger influence on spheroid formation than Butyl-LBA, because the MBA ligand has a stronger interaction with HepG2 cells than the LBA ligand. Such different inhibition effects on spheroid formation could be caused by the difference in binding affinity of carbohydrate ligands to the ASGPRs. Notably, 50 mM Butyl-LBA and 300 mM GalNAc resulted in cell aggregates with no significant difference in circularity and the aspect ratio, indicating that these two solutions exhibit a similar inhibiting effect on the formation of HepG2 spheroids. Furthermore, 100 mM Butyl-LBA showed stronger inhibition, with the HepG2 cells assembled into looser cell aggregates with a lower circularity (0.38) and a higher aspect ratio (1.84) than cell aggregates formed in the presence of 50 mM Butyl-LBA. However, no significant changes in circularity and the aspect ratio were observed when increasing Butyl-MBA from 50 to 100 mM. Moreover, the morphology of cell aggregates with 100 mM Butyl-LBA was similar to that with 100 mM Butyl-MBA, revealing that the inhibiting effect of spheroid formation is almost identical at a higher inhibitor concentration (Figure S26). Finally, HepG2 cells remained dispersed and failed to form cell aggregates at a concentration of 300 mM for either Butyl-LBA or Butyl-MBA (Figure S27), indicating that the aggregation of cells on carbohydrate gels is completely suppressed in the presence of these inhibitors.

### Cell Migration on Carbohydrate Hydrogels

**Figure 6:**
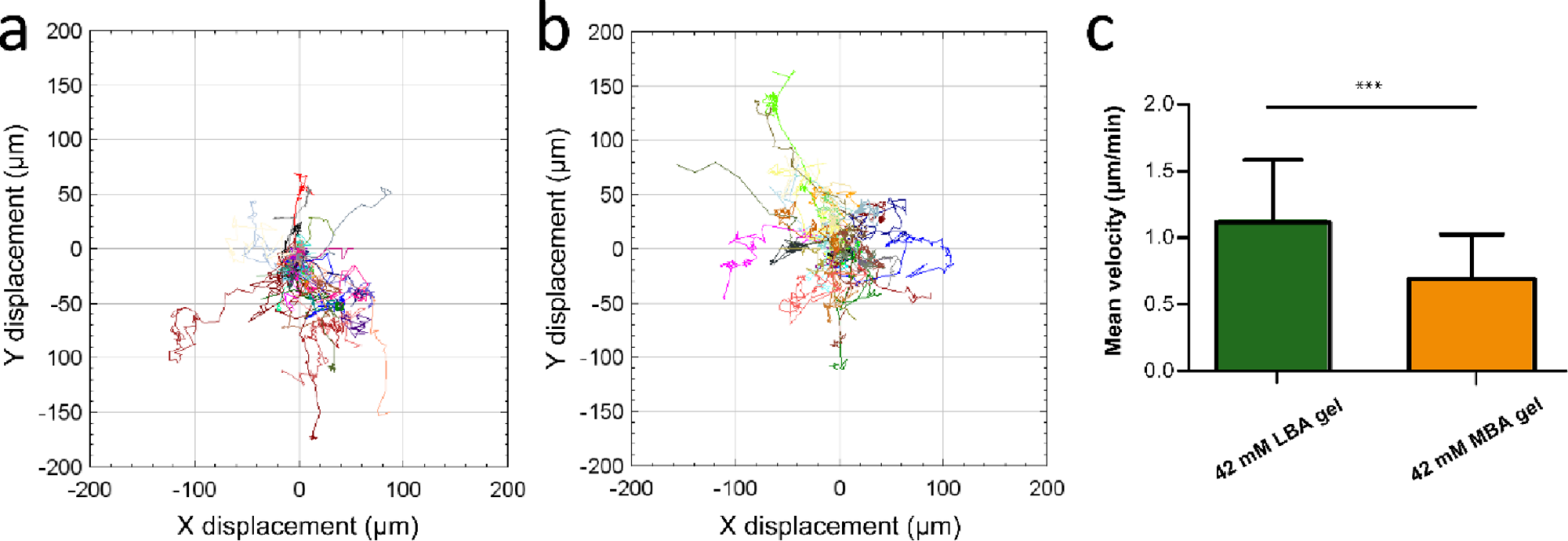
Cell migration on carbohydrate amphiphile gels. Representative trajectory plots of HepG2 cells on 42 mM LBA gel (a) and 42 mM MBA gel (b). (c) Quantitative analysis of average migration speed of HepG2 cells on two different gels. The trajectory and mean velocity are calculated by Trackmate in ImageJ. For each group, *n* = 20. Only tracks of single cells were included. The statistical analysis of cell migration velocity was determined using an unpaired two-sided *t*-test. *P* values of statistical significance are represented as * *P* < 0.05, ** *P* < 0.01, *** *P* < 0.001, N.S., not significant.

The formation of liver spheroids is a synergistic effect of cell adhesion, migration, and proliferation.^20,49^ On LBA or MBA gels, the carbohydrate ligands enhance the ASGPR-dependent cell-adhesion to facilitate the coassembly of hepatocytes and result in a gradual aggregation to form multicellular aggregates. To investigate whether the spheroid variations in diameter and spheroid formation efficiency can be attributed to differences in cell motility between different carbohydrate gel surfaces, live-cell imaging microscopy was employed to visualize and track cell migration and aggregation, especially in the early stages of spheroid formation. To analyze cell migration parameters before spheroid formation, including cell migration trajectories and average cell velocity, the migration of 15–25 representative cells on 42 mM gels was measured during the first 450 min after seeding. As shown in Figure 6a,b, the HepG2 cells migrated over long distances of up to ∼200 μm on the carbohydrate gels. Despite high variability in the migration distances, it is clear that, on 42 mM LBA gels, cells migrated faster (1.12 μm/min) than on 42 mM MBA gels (0.69 μm/min), as indicated in Figure 6c. The observed relative migration speeds are in line with the faster migration on higher stiffness substrates reported in the literature.^56,57^ We speculate that the ligand type may also contribute to the difference in cell migration since the MBA ligand exhibits a stronger interaction with HepG2 cells, as was shown in the competition experiments. Therefore, the physical and biological parameters of carbohydrate-based supramolecular hydrogels, including gel stiffness and the ligand type, significantly affect cell migration speed.

## Conclusions

In summary, we have developed two fully synthetic supramolecular hydrogels that do not require functionalization with a peptide adhesion motif or chemical cross-linking to mimic the fibrous architecture, dynamicity, and bioactivity of ECMs. The self-assembled hydrogels not only provide a promising platform to elucidate the role of carbohydrate ligand structure, gel stiffness, and ligand density on spheroid formation but also offer a new avenue toward a processible and bioactive mimic of the natural microenvironment for liver tissue engineering. In future work, chemical cross-linking between fibers may be incorporated to gain better control over gel stiffness and to introduce strain-stiffening behavior to achieve a full mimic of ECMs.

## Supporting Information Available

Experimental details of CMC determination, self-assembled morphologies, fitting of SAXS data, gel preparation and rheological measurement, bundle domains in LBA and MBA amphiphile gels, cell culture, and spheroid characterization (PDF)
