# Supplementary material for: Hepatic Spheroid Formation on Carbohydrate-Functionalized Supramolecular Hydrogels
Source: Biomacromolecules. 2023 May 29;24(6):2447–58. doi: 10.1021/acs.biomac.2c01390 (PMC10265662; doi:10.1021/acs.biomac.2c01390)
Supplement: Supplementary file 1 — bm2c01390_si_001.pdf [file bm2c01390_si_001.pdf]

Supporting information

Hepatic Spheroid Formation on Carbohydrate-Functionalized Supramolecular Hydrogels

Jie Liu[1], Ying Zhang[2], Kim van Dongen[3], Chris Kennedy[4], Maaike J.G. Schotman[2], Patricia P. Marín San Román[1], Cornelis Storm[4], Patricia Y.W. Dankers[2], and Rint P. Sijbesma*[1]*

[1]Institute for Complex Molecular Systems, Department of Chemical Engineering and Chemistry, Eindhoven University of Technology, 5600 MB, Eindhoven, The Netherlands.

[2]Institute for Complex Molecular Systems, Department of Biomedical Engineering, Eindhoven University of Technology, 5600 MB, Eindhoven, The Netherlands.

[3]CytoSMART Technologies B.V., Vrijstraat 9B, 5611 AT Eindhoven, The Netherlands.

[4]Institute for Complex Molecular Systems, Department of Applied Physics, Eindhoven University of Technology, 5600 MB, Eindhoven, the Netherlands.

Contents

1. Instruments 3
2. Materials 3
3. Synthetic procedures....................3
3.1. LBA amphiphile 3
3.2. MBA amphiphile 7
3.3. LBA-butyl amide....................8
3.4. MBA-butyl amide....................9
4. Supplementary results....................11
4.1. CMC determination 11
4.2. Self-assembled morphologies....................12
4.3. Fitting of SAXS profiles....................13
4.4. Gel preparation and rheological measurement 14
4.5. Bundle domains in LBA and MBA amphiphile gels....................14
4.6. Cell culture and spheroids characterization....................16
4.7. Cell culture experiments in the presence of Butyl-LBA and Butyl-MBA 21
References 22

1. Instruments

^{1}H NMR and ^{13}C NMR spectra were recorded on a 400 MHz NMR (Varian Mercury Vx or Varian 400MR) operating at 400 MHz for ^{1}H NMR and 100 MHz for ^{13}C NMR. Chemical shift (δ) is reported in parts per million (ppm) from tetramethylsilane (TMS) or using resonance of the deuterated solvent as internal standard. Spiliting patterns are labelled as singlet (s), doublet (d), double doublet (dd), triplet (t), quartet (q) and multiplet (m). Liquid chromatography-mass spectrometry (LC-MS) was performed on a Thermo Fisher Scientific LCQ Fleet ESI-MS with H_2O (0.1% formic acid) as eluents.

2. Materials

All starting materials were commercially available and were used without further purification unless noted otherwise. Deuterated solvents were purchased from Cambridge Isotopes Laboratories. Reactions were carried out under inert argon atmosphere and all glassware was dried in an oven before the reaction. Lactobionic acid (LBA), 1,10-diaminodecane, N-acetylgalactosamine (GalNAc) and n-butylamine were purchased from Sigma-Aldrich, and hexamethylene diisocyanate (HDI) was purchased from Tokyo Chemical Industry (TCI). Maltobionic acid (MBA) was purchased from Biosynth Carbosynth.

3. Synthetic procedures

3.1. LBA amphiphile

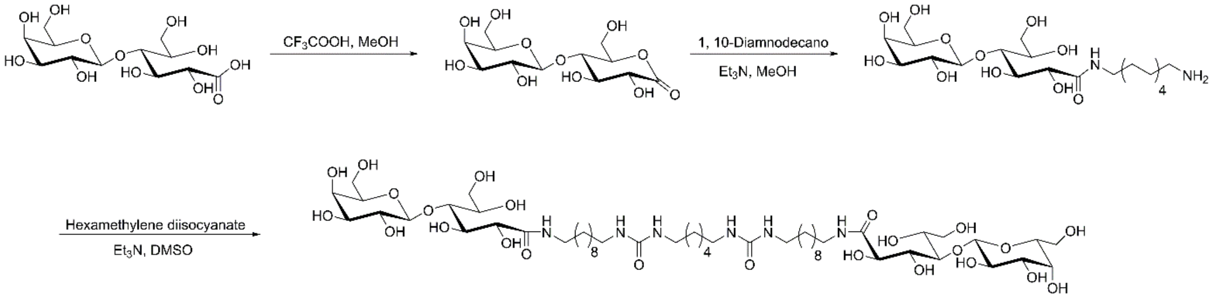

Scheme S1. Synthetic route for LBA amphiphile.

Lactobiono-δ-lactone

The literature procedure[1] was modified as follows: 10.0 g (27.9 mmol) of lactobionic acid was dissolved in 100 mL of dry methanol, and 0.5 mL trifluoroacetic acid (TFA) was added and the reaction mixture was stirred at 50 °C under argon atmosphere. After 3 h, solvent was evaporated under reduced pressure, the reaction was continued after re-dissolving the reaction mixture in methanol and another 0.5 mL of TFA was added. The above step was repeated 3 times to provide lactobiono-δ-lactone as a white, foamy solid. The product was used without further purification.

LBA-C10-NH$_2$

3.0 g (1.0 eq., 8.82 mmol) of lactobiono-δ-lactone was dissolved in 100 mL of methanol, and then 4.55 g (3.0 eq., 26.5 mmol) of 1, 10-decanodiamine and 1.23 mL (1.0 eq., 8.82 mmol) of triethylamine were added. The reaction was carried out at 50 °C under argon atmosphere for 24 h. Most of the methanol was removed, and the resulting concentrated solution was added dropwise into 500 mL of a 4:1 chloroform/diethyl ether mixture to provide a white precipitate.

This solid was washed twice with 200 mL of chloroform. LBA-C10-NH_2 (3.52 g) was obtained after vacuum filtration and drying *in vacuo* in 87% yield, containing a small amount of di-lactobionic amides as byproducts without further purifications.

^{1}H NMR (400 MHz, *d6*-DMSO) δ ppm: 7.55 (t, 1H), 4.27 (d, 1H), 4.08 (d, 1H), 4.00 (m, 1H), 3.74-3.14 (m, 18H from carbohydrate protons), 3.16-2.98 (m, 2H), 2.54 (m, 2H), 1.47-1.08 (m, 16H). ^{13}C NMR (400 MHz, *d6*-DMSO) δ ppm: 172.51, 105.07, 83.42, 79.65, 76.17, 73.70, 72.43, 71.87, 71.58, 70.92, 68.68, 62.82, 61.11, 41.87, 40.88, 40.59, 40.39, 40.18, 39.97, 39.76, 39.55, 39.34, 38.74, 33.23, 29.66, 29.45, 29.42, 29.24, 26.85, 26.79. ESI-MS m/z [M+H] calcd for $C_{22}H_{45}N_2O_{11}^+$ 513.50, found 513.29.

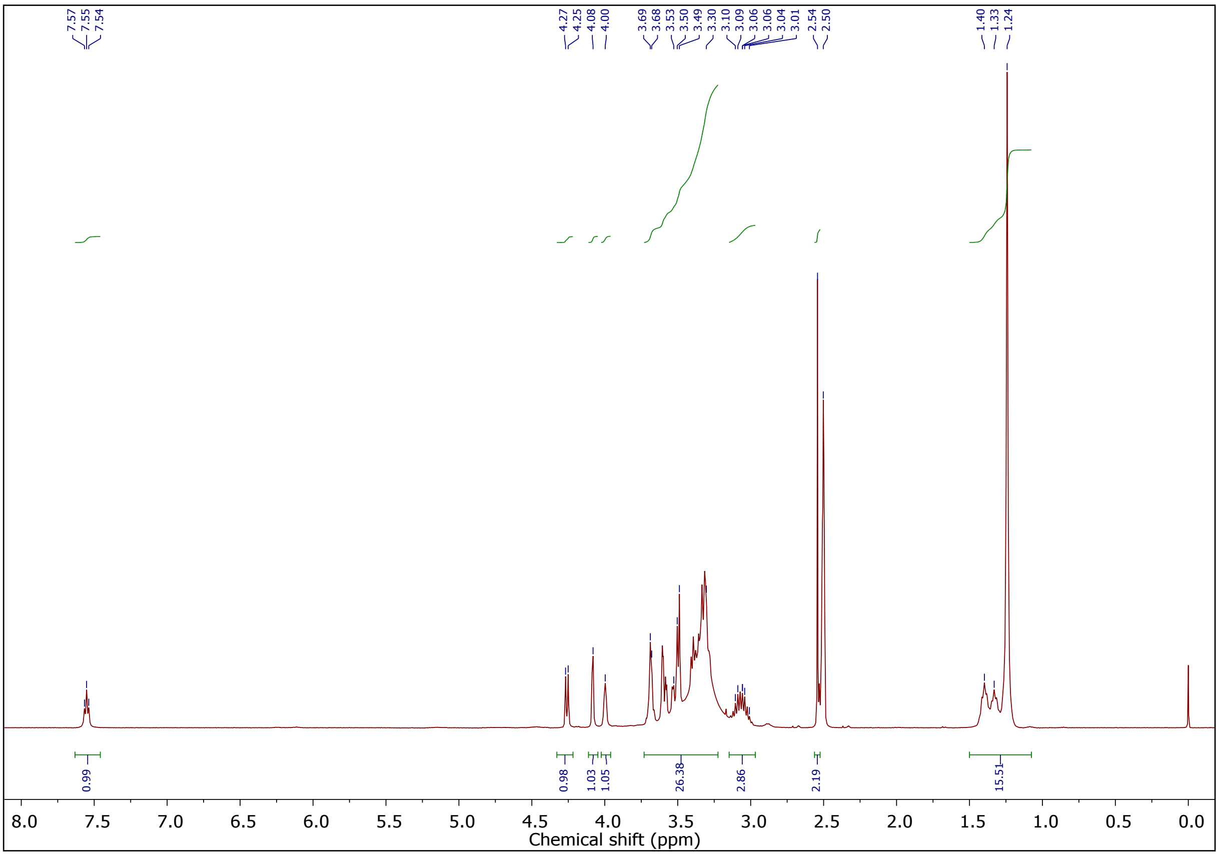

Figure S1. ^{1}H NMR spectrum of LBA-C10-NH_2 in *d6*-DMSO.

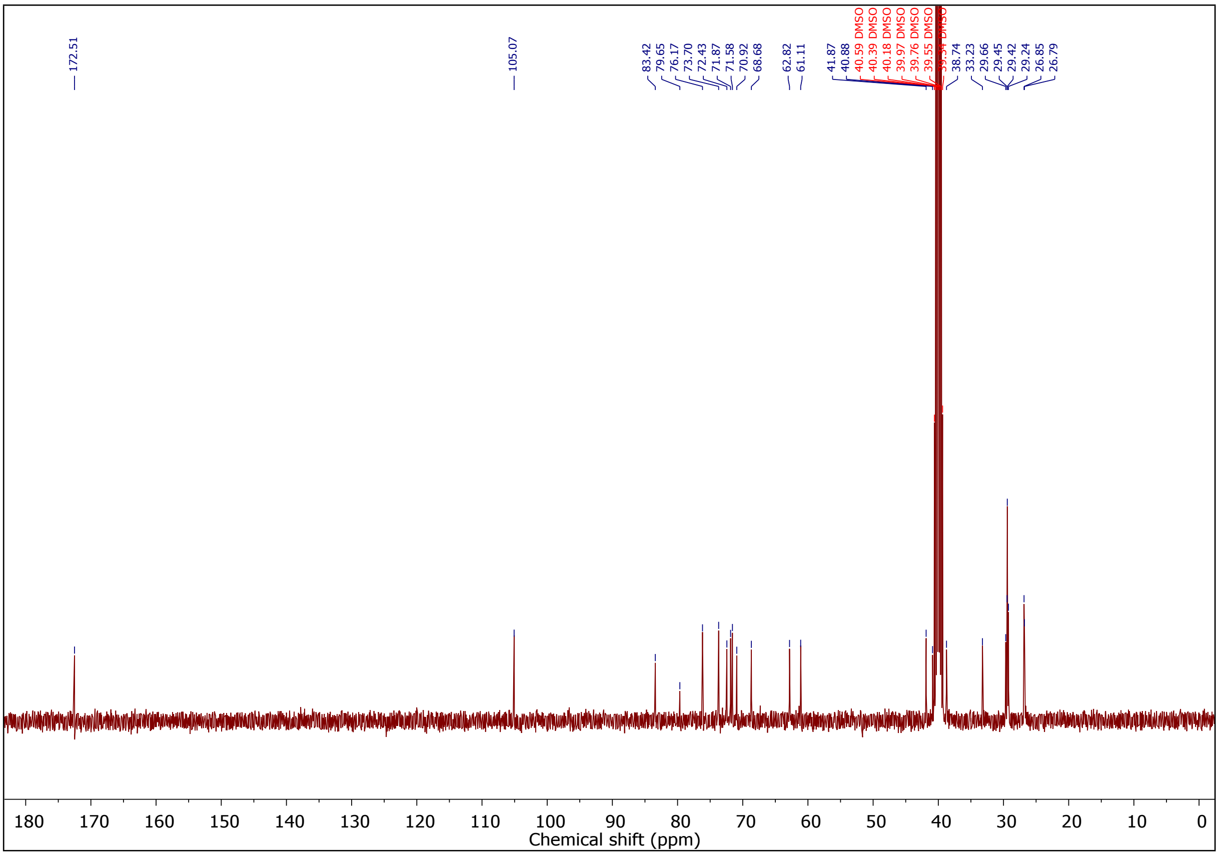

Figure S2. ^{13}C NMR spectrum of LBA-C10-NH_2 in *d6*-DMSO.

LBA amphiphile

1.5 g (2.93 mmol) of LBA-C10-NH_2 was dissolved in 10 mL of dry DMSO, together with 1.0 eq. of triethylamine. To this solution, 246 mg (1.46 mmol) of hexamethylene diisocyanate in 10 mL of dry DMSO was added dropwise over 1 h, and the reaction mixture was stirred vigorously overnight at room temperature under argon atmosphere. After this time, the reaction solution was added dropwise into 500 mL of chloroform/diethyl ether (2:1) to provide crude product as a white solid after vacuum filtration. Relatively pure (> 95%) LBA amphiphile was obtained by recrystallization from MeOH with a yield of ~60%.

^{1}H NMR (400 MHz, *d6*-DMSO) δ ppm: 7.55 (t, 2H), 5.71 (m, 4H), 5.16-5.10 (m, 4H), 4.77 (m, 4H), 4.67 (t, 2H), 4.46 (m, 4H), 4.27 (d, 2H), 4.11 (m, 2H), 4.00 (m, 4H), 3.74-3.64 (m, 4H), 3.63-3.56 (m, 4H), 3.56-3.45 (m, 6H), 3.42-3.37 (t, 2H), 3.37-3.26 (m, 4H, overlap with H_2O), 3.14-2.98 (t, 4H), 2.98-2.86 (m, 8H), 1.55-1.13(m, 40H). ^{13}C NMR (400 MHz, *d6*-DMSO) δ ppm: 172.50, 158.57, 105.07, 83.42, 76.17, 73.69, 72.44, 71.87, 71.58, 70.91, 68.70, 62.82, 61.14, 38.78, 30.52, 29.69, 29.51, 29.46, 29.30, 29.28, 26.89, 26.85, 26.60. ESI-MS m/z [M+H] calcd for $C_{52}H_{101}N_6O_{24}^+$ 1193.68, found 1193.33.

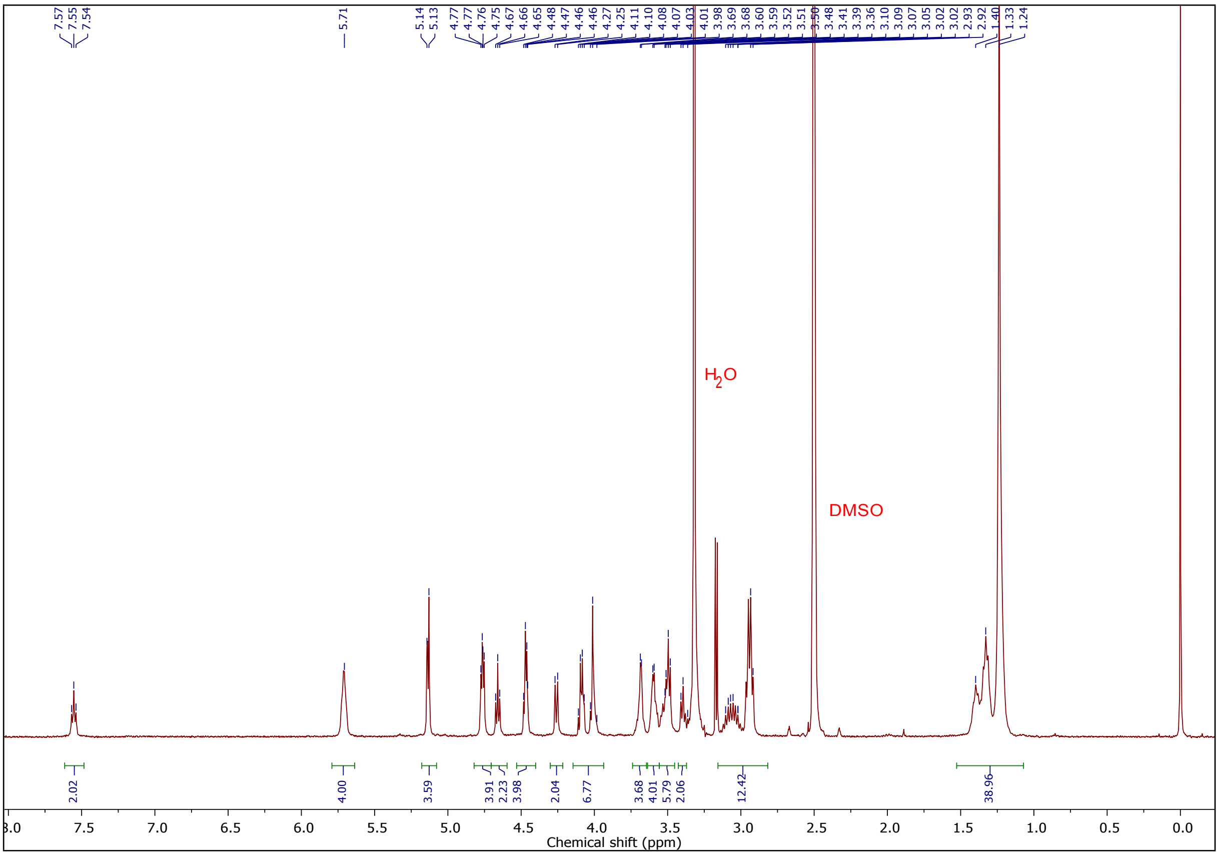

Figure S3. ^{1}H NMR spectrum of LBA amphiphile in *d6*-DMSO.

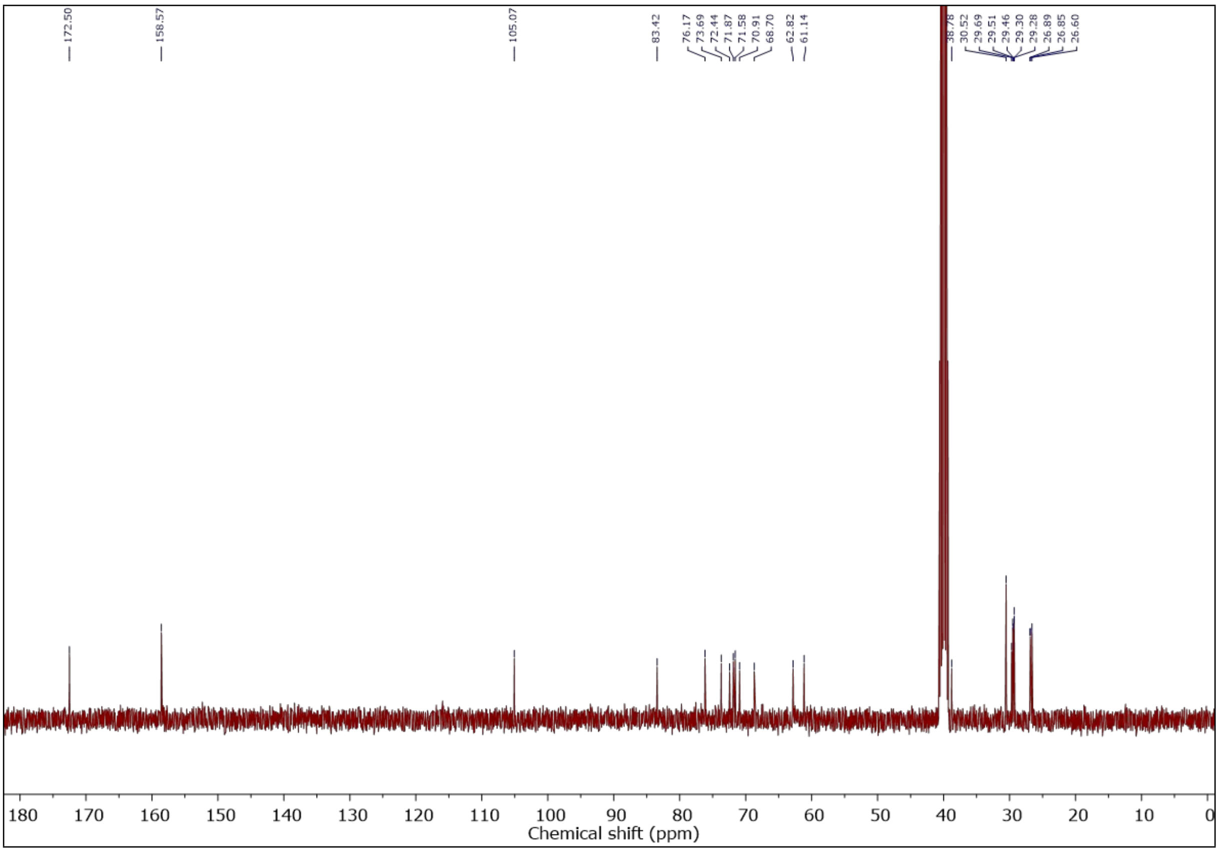

Figure S4. ^{13}C NMR spectrum of LBA amphiphile in *d6*-DMSO.

3.2. MBA amphiphile

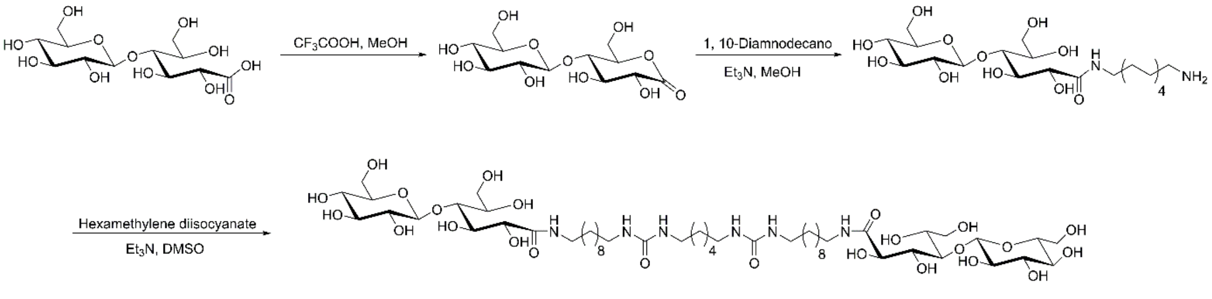

Scheme S2. Synthetic route for MBA amphiphile.

The synthesis of MBA amphiphile was performed following the procedures for LBA amphiphile and gave the product in a yield of 67%.

^{1}H NMR (400 MHz, *d6*-DMSO) δ ppm: 7.55 (t, 2H), 5.72 (m, 4H), 5.53 (d, 2H), 5.33 (d, 2H), 4.89 (m, 6H), 4.67 (d, 2H), 4.54-4.40 (m, 6H), 4.04-3.92 (m, 4H), 3.70-3.35 (m, 16H), 3.28-3.20 (m, 2H), 3.14-2.98 (m, 6H), 2.99-2.88 (m, 8H), 1.46-1.14 (m, 40H). ^{13}C NMR (400 MHz, *d6*-DMSO) δ ppm: 172.38, 158.56, 101.20, 83.48, 73.81, 73.64, 72.75, 72.56, 72.41, 72.06, 70.35, 63.06, 61.11, 40.88, 38.75, 30.52, 29.71, 29.52, 29.46, 29.30, 29.27, 26.88, 26.83, 26.60. ESI-MS m/z [M+H] calcd for $C_{52}H_{101}N_6O_{24}^+$ 1193.68, found 1193.42.

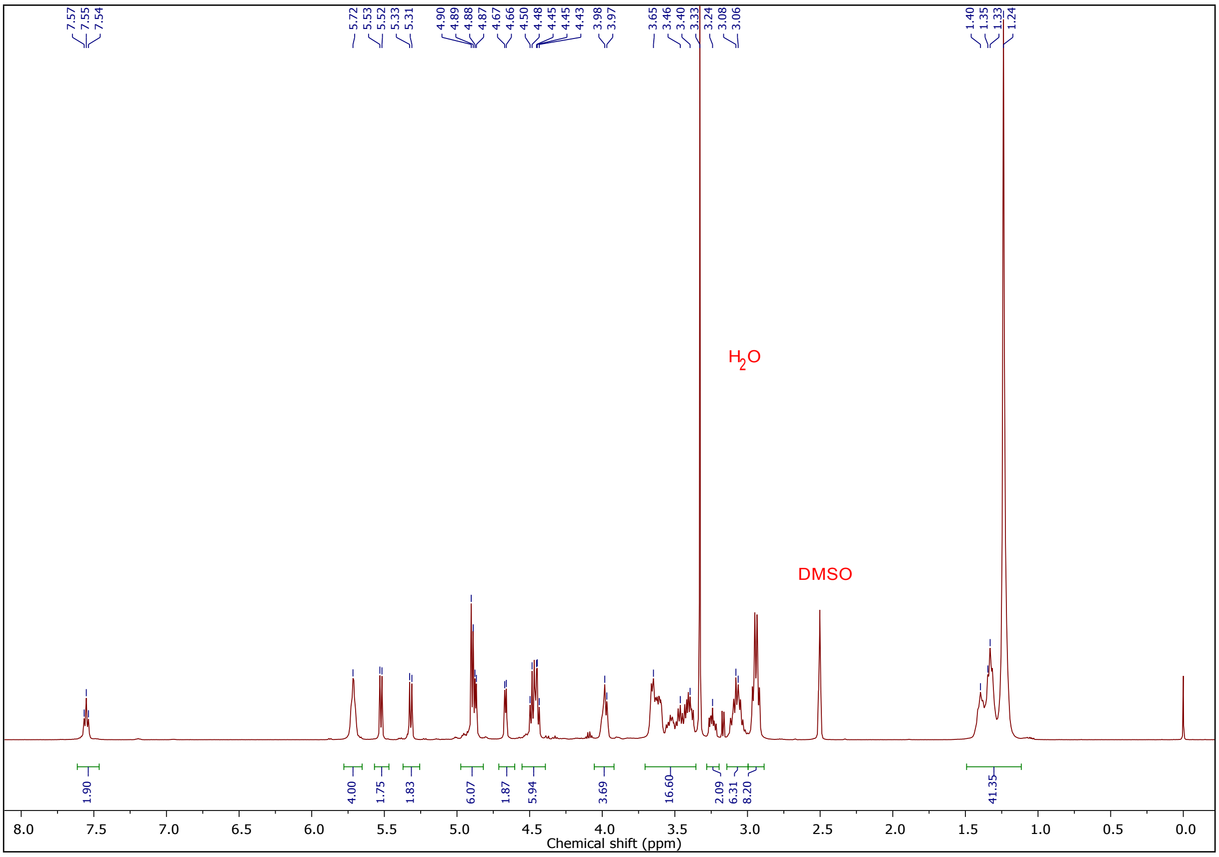

Figure S5. ^{1}H NMR spectrum of MBA amphiphile in *d6*-DMSO.

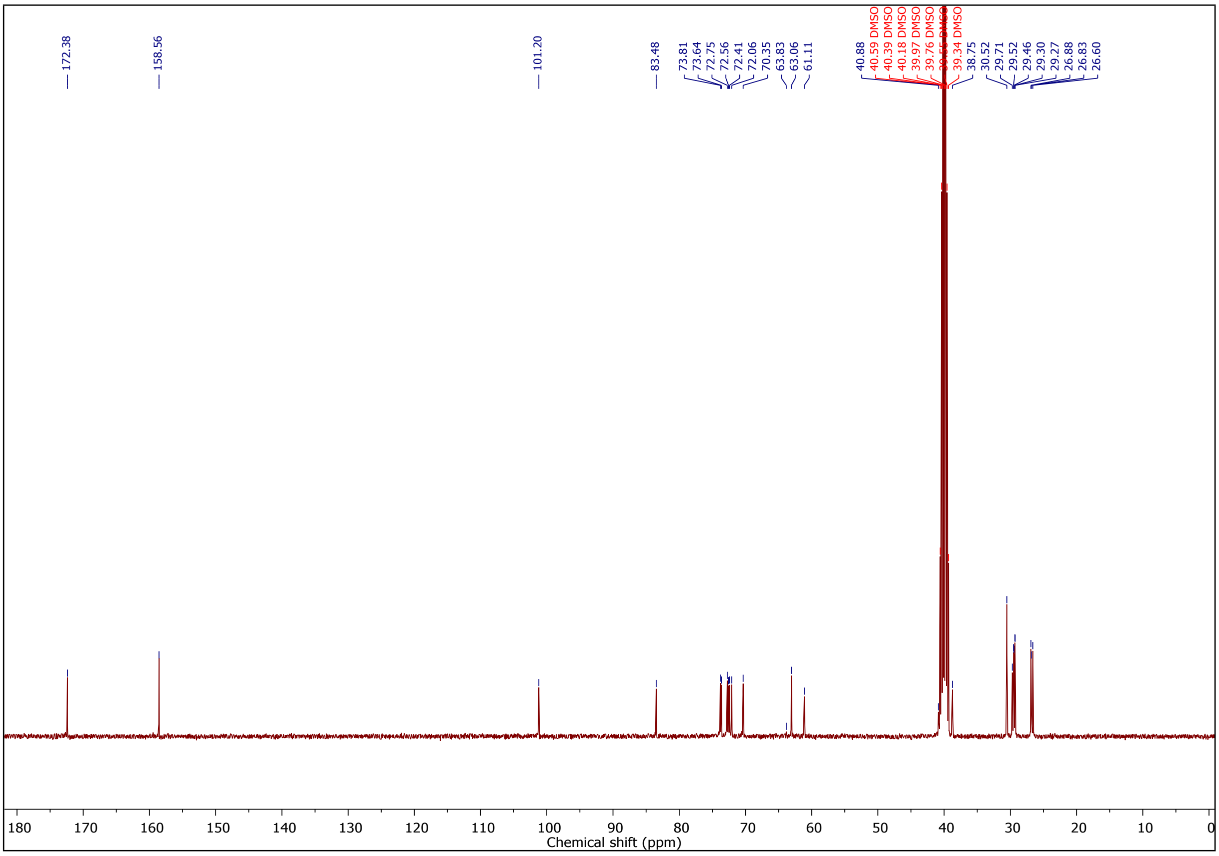

Figure S6. ^{13}C NMR spectrum of MBA amphiphile in *d6*-DMSO.

3.3. LBA-butyl amide

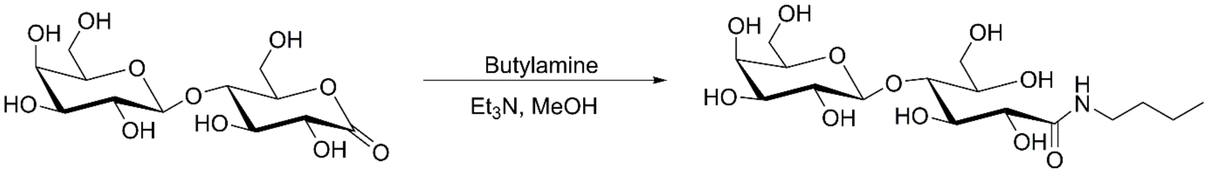

Scheme S3. Synthetic route for LBA-butyl amide.

300 mg (1.0 eq., 0.882 mmol) of lactobiono-δ-lactone was dissolved in 5 ml of ethanol, and then 97 mg (1.5 eq., 1.32 mmol) of n-butylamine and 133 mg (1.5 eq., 1.32 mmol) of triethylamine were added. After stirring at room temperature overnight, the reaction mixture was added dropwise into 50 mL of diethyl ether to remove excess butylamine and triethylamine. A white precipitate was collected by centrifugation and further purified by recrystallization from ethanol to provide 110 mg of a white solid (30% yield) after drying in vacuo.

^{1}H NMR (400 MHz, MeOD) δ ppm: 4.48 (d, 1H), 4.32 (d, 1H), 4.21 (q, 1H), 3.45-4.00 (H from carbohydrate protons, including H from residual EtOH solvent), 3.24 (t, 2H), 1.51 (t, 2H), 1.38 (t, 2H), 0.94 (t, 3H).

ESI-MS m/z [M+H] calcd for $C_{16}H_{32}NO_{11}^{+}$ 414.19, found 414.00.

3.4. MBA-butyl amide

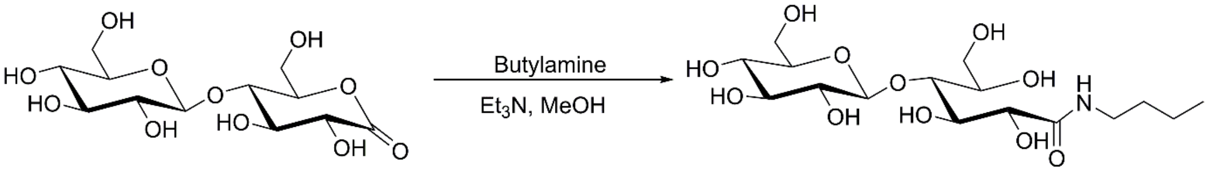

Scheme S4. Synthetic route for MBA-butyl amide.

The synthesis of MBA-butyl amide was performed following the procedures for LBA butyl amide and gave the product in a yield of 44% (ca. 160 mg).

^{1}H NMR (400 MHz, MeOD) δ ppm: 5.06 (d, 1H), 4.23 (d, 1H), 4.18 (q, 1H), 3.55-3.97 (H from carbohydrate protons, including H from residual EtOH solvent), 3.45 (q, 1H), 3.24 (t, 2H), 1.51 (t, 2H), 1.38 (t, 2H), 0.94 (t, 3H). ESI-MS m/z [M+H] calcd for $C_{16}H_{32}NO_{11}^{+}$ 414.19, found 414.00.

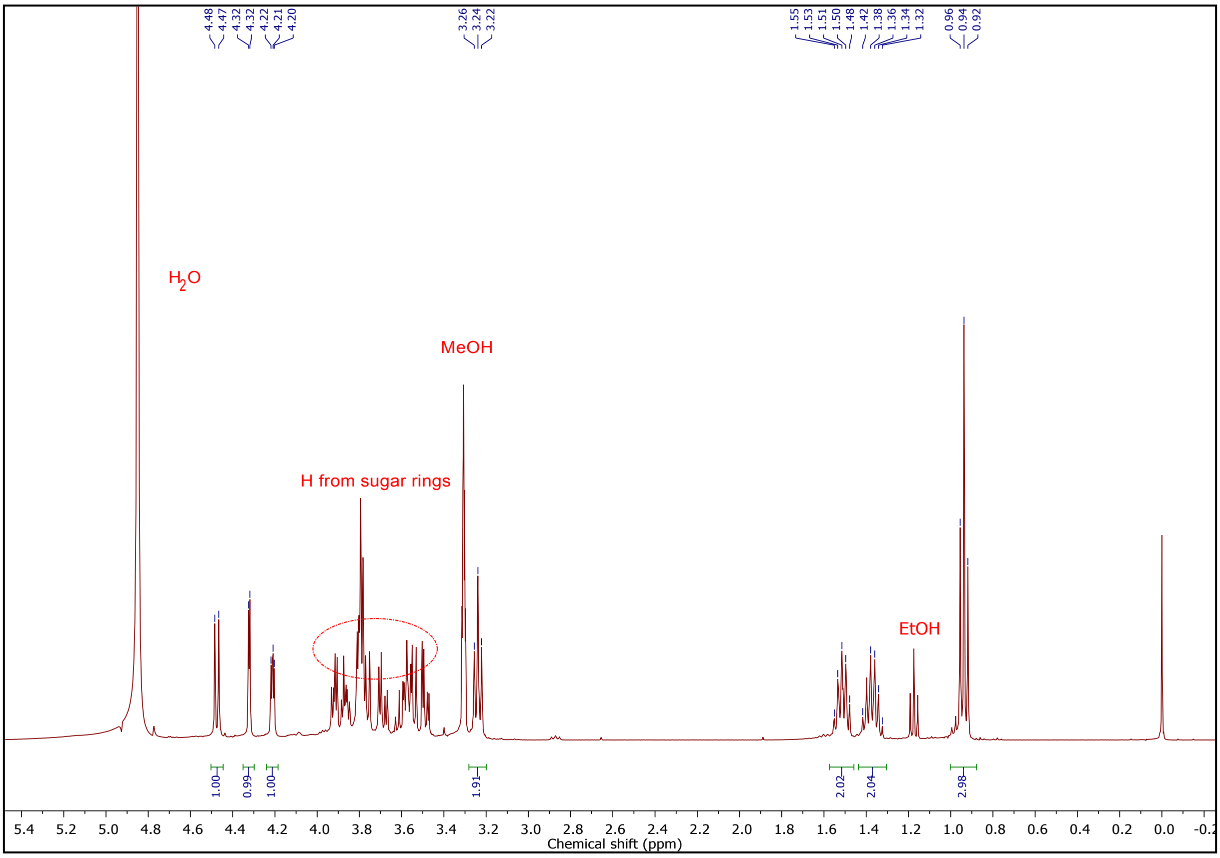

Figure S7. ^{1}H NMR spectrum of LBA-butyl amide in MeOD.

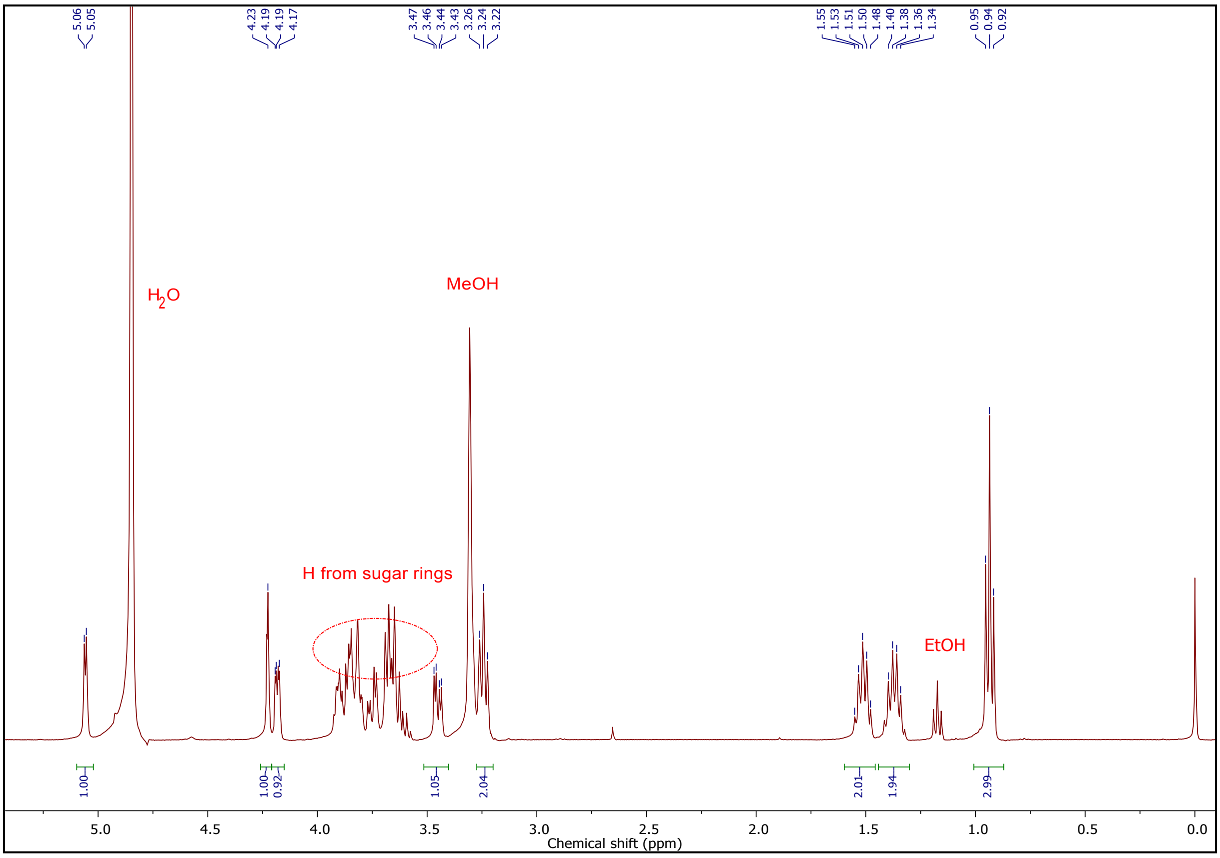

Figure S8. ^{1}H NMR spectrum of MBA-butyl amide in MeOD.

4. Supplementary results

4.1. CMC determination

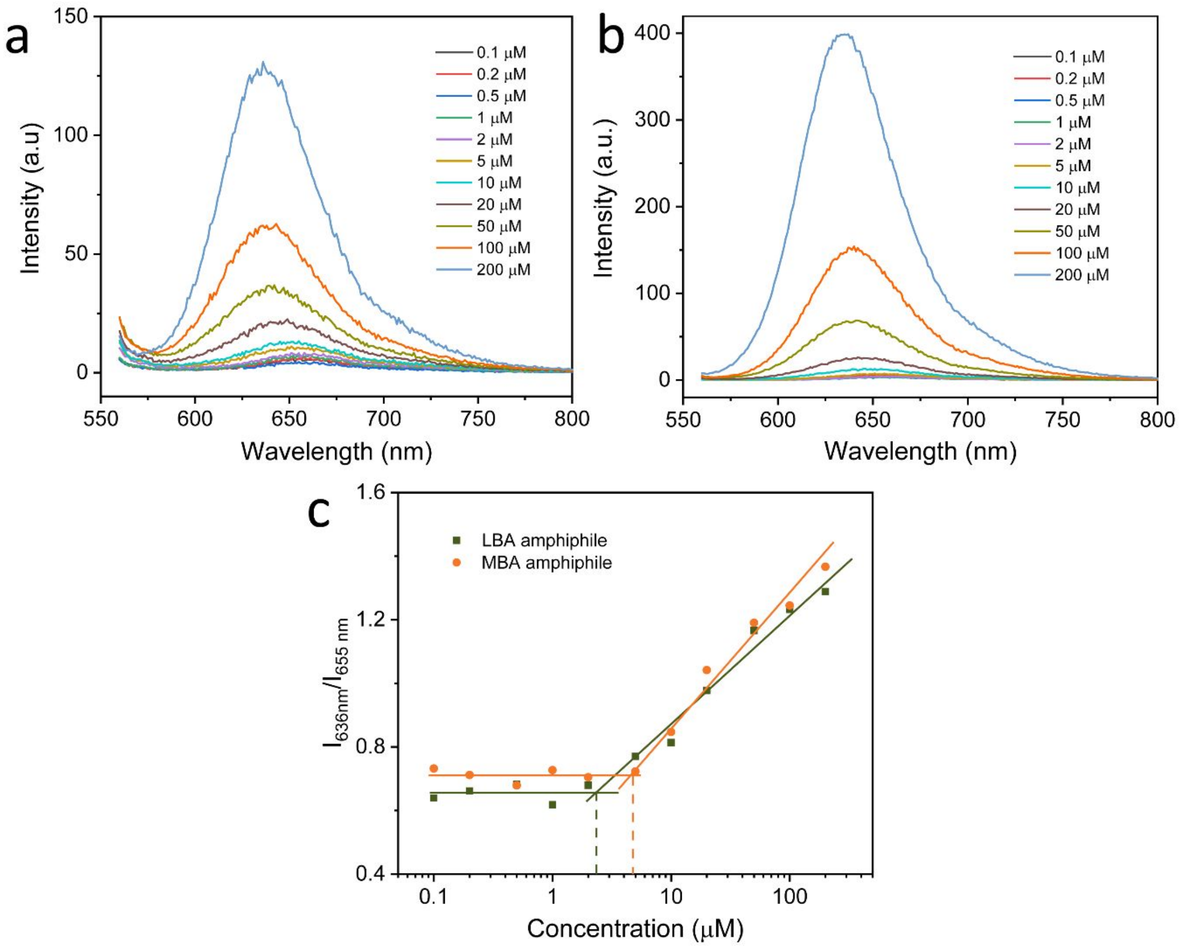

Figure S9. CMC determination of two carbohydrate amphiphiles. Emission spectra of Nile red solution (1.0 μM) in water with different concentration of (a) LBA amphiphile and (b) MBA amphiphile. (c) Plots of Nile red fluorescence intensity ratio (I_{636nm}/I_{655nm}) versus log concentration of the LBA and MBA amphiphiles at room temperature.

4.2. Self-assembled morphologies

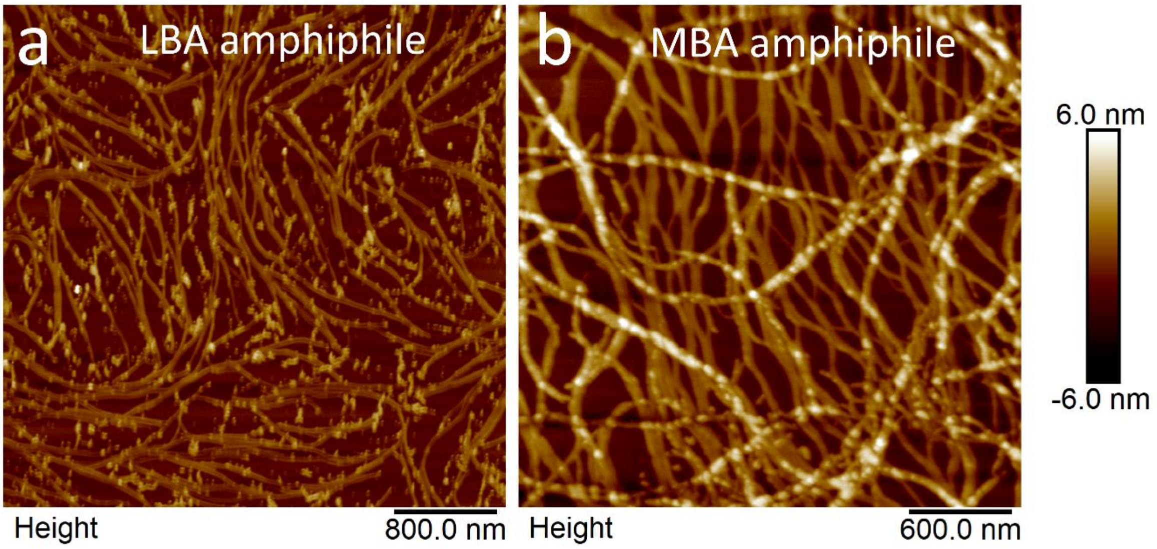

Figure S10. Representative zoom-in AFM height images of LBA amphiphile (a) and MBA amphiphile (b) at 0.5 mM in water, respectively.

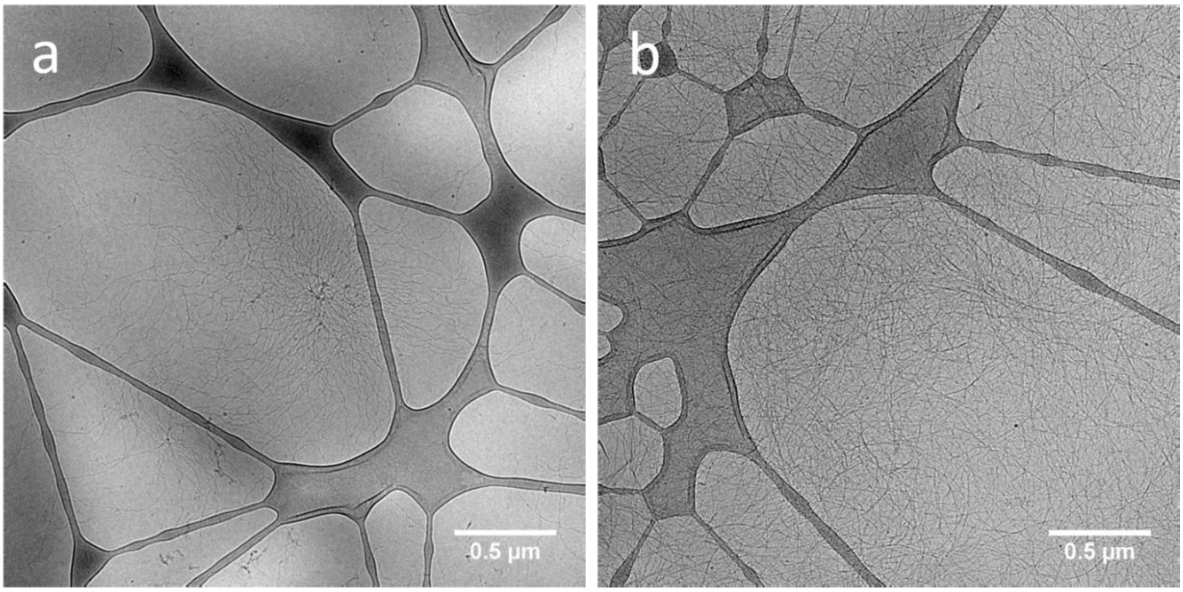

Figure S11. Representative zoom-out cryo-TEM images of LBA amphiphile in water at 0.1 mM (a) and 0.5 mM (b), respectively.

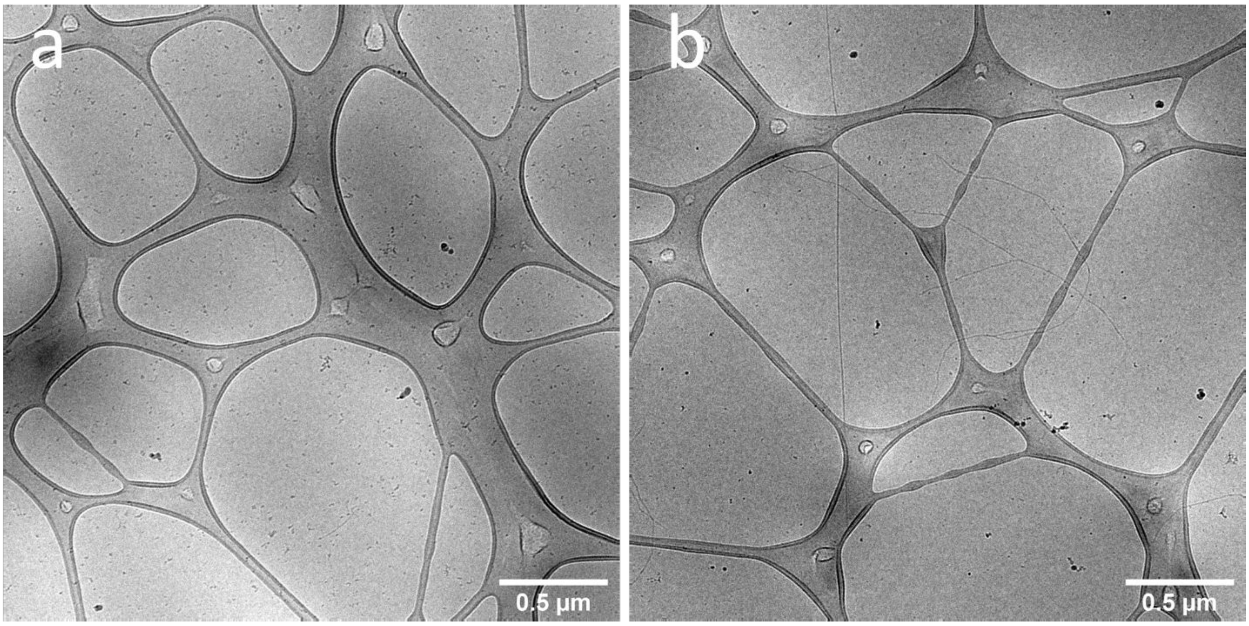

Figure S12. Representative zoom-out cryo-TEM images of MBA amphiphile in water at 0.1 mM (a) and 0.5 mM (b), respectively.

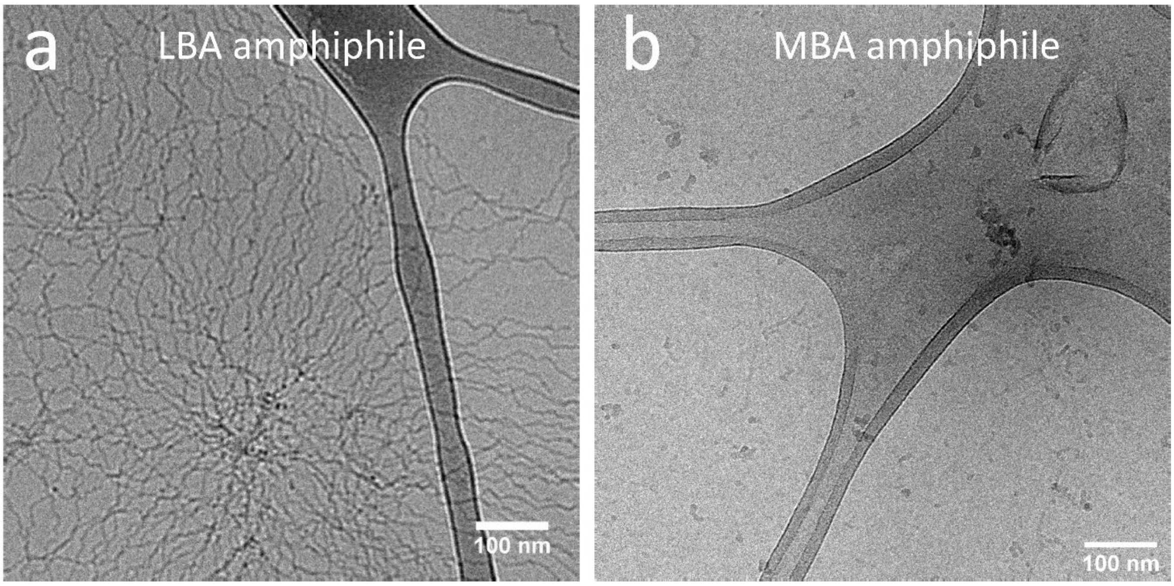

Figure S13. Representative zoom-in cryo-TEM images of LBA amphiphile (a) and MBA amphiphile (b) at 0.1 mM in water, respectively.

4.3. Fitting of SAXS profiles

Small-angle X-ray scattering was used to determine the cross-sectional radius of amphiphile fibers in solution. The values of water density (0.99820 g/cm^3), amphiphiles solution density (0.99960 g/cm^3), scattering length density of water (sld. solvent) and scattering length density of amphiphile (sld or sld. core) are adopted from previous work in our group.[2] The fitting parameters are shown in the **Table S1**.

Table S1. Fitting parameter in SAXS profiles of LBA and MBA amphiphiles at 1.0 wt% (8.4 mM) solution using a cylindrical form factor.

**Parameters**	**LBA amphiphile**		**MBA amphiphile**	
	Value	Error	Value	Error
**Scale**	0.00238	1.456×10^{-7}	0.00255	1.20×10^{-7}
Background	5.29×10^{-4}	1.98×10^{-8}	3.35×10^{-4}	1.43×10^{-8}
Sld/Ang^{-2}	10.6×10^{-6}			
Sld solvent/Ang^{-2}	9.37×10^{-6}			
Radius/Ang	33.6	3.14×10^{-4}	33.9	4.39×10^{-4}
Length/Ang	10000 (fixed value)			
Distribution of radius	0.108	1.17×10^{-5}	0.148	9.43×10^{-3}
Reduced Chi^2	1.14×10^{6}		9.90×10^{5}	

4.4. Gel preparation and rheological measurement

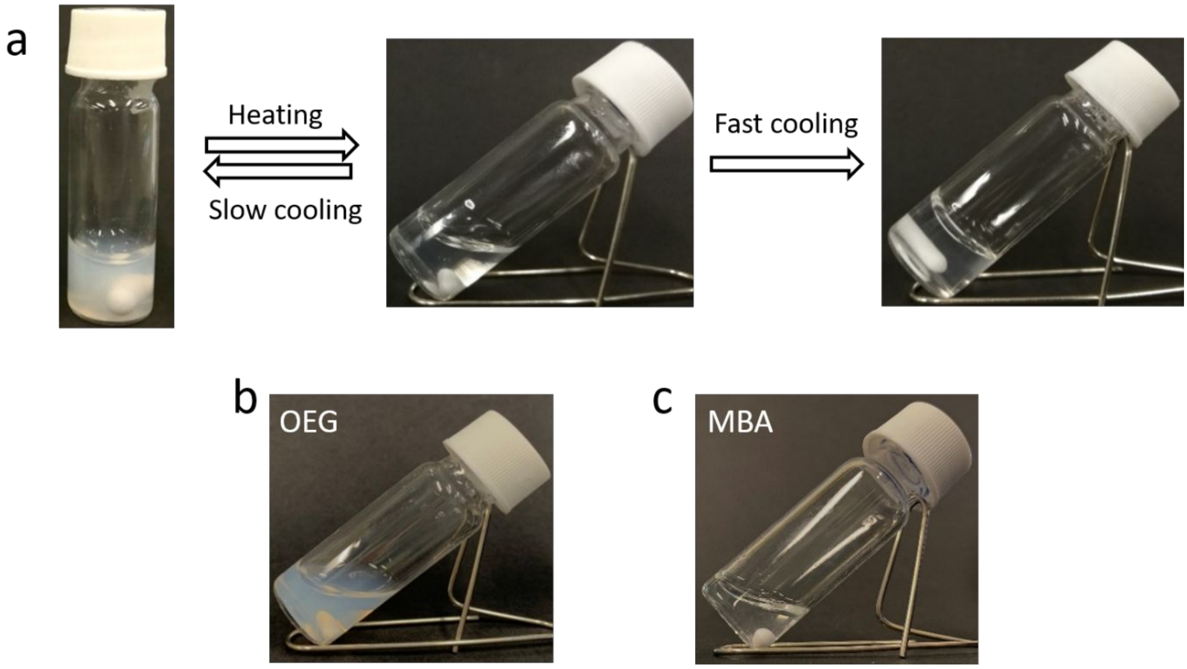

Figure S14. (a) LBA amphiphile hydrogel preparation from turbid aqueous solution at 17 mM; hydrogels cannot be formed at below 40 mM for OEG amphiphile (b), and 20 mM for MBA amphiphile (c).

4.5. Bundle domains in LBA and MBA amphiphile gels

For the LBA and MBA amphiphiles, transparent solution or gels were only obtained with a heating-fast cooling cycle. Upon slow cooling, however, the amphiphiles formed opaque solutions (gels) or precipitates due to formation of large crystalline structures. These competing,

parallel pathways are also found in many LMWGs[3,4], and it is clear that crystallization can be suppressed by fast cooling to promote nanofiber growth. 4.5. Bundle domains in LBA and MBA amphiphile gels

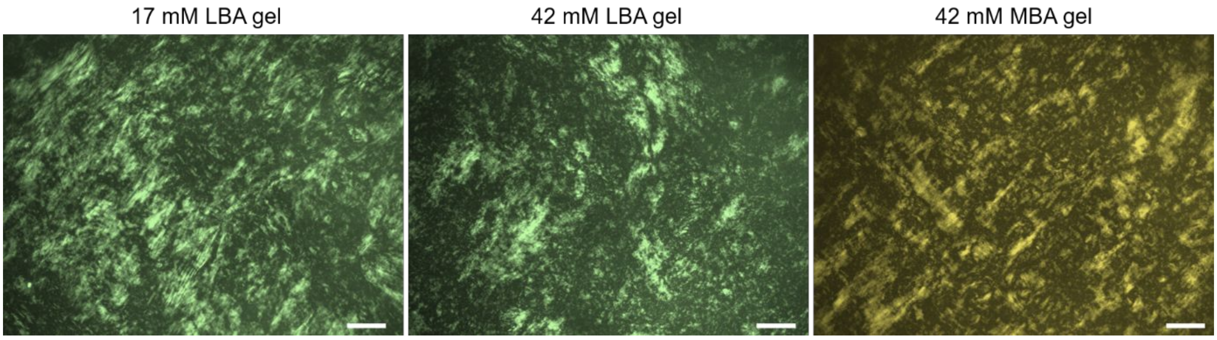

Figure S15. Polarized optical microscopy images of three carbohydrate gels. The scale bar is 500 μm.

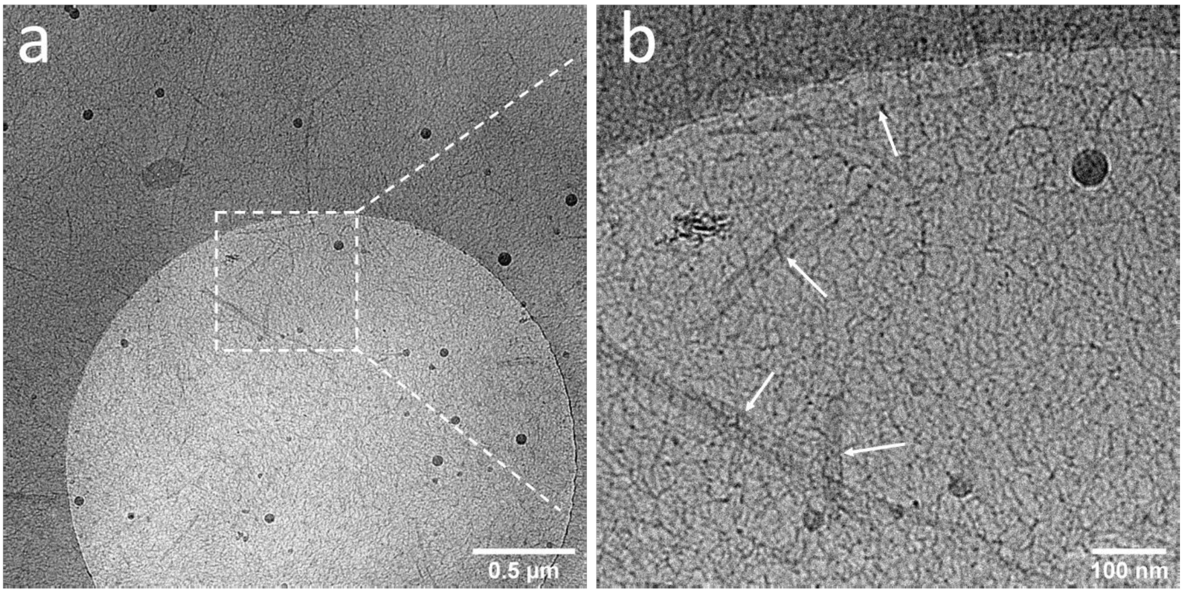

Figure S16. Representative cryo-TEM images of 17 mM LAB amphiphile hydrogel in water at after heating-rapid cooling (a: zoom out and b: zoom in). Fibers and bundle structures were both observed (bundles are marked by arrows).

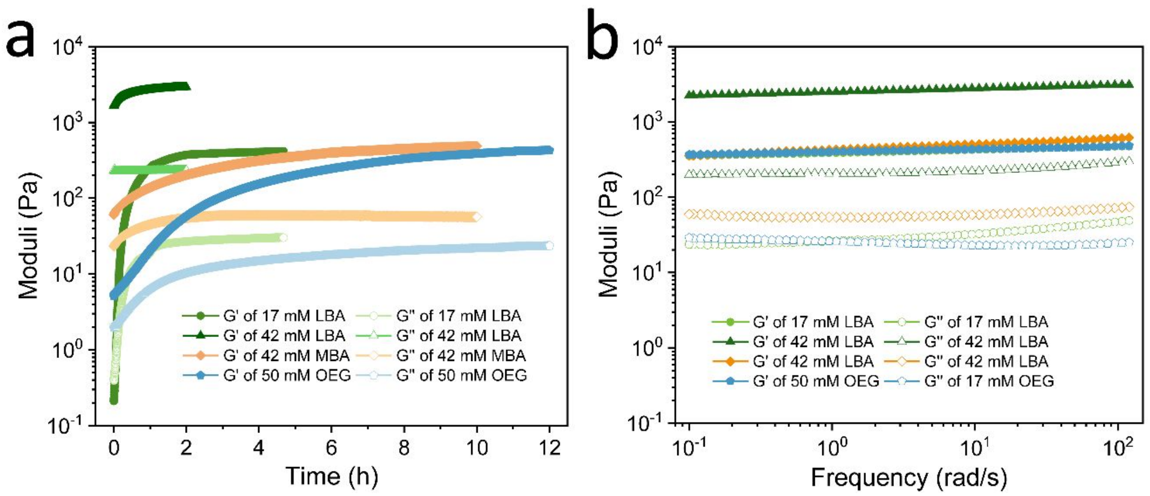

Figure S17. Time and frequency sweep oscillatory shear rheology for LBA and MBA amphiphile hydrogels with a strain of 1.0%. The measurement temperature was set up at 37 °C

4.6. Cell culture and spheroids characterization

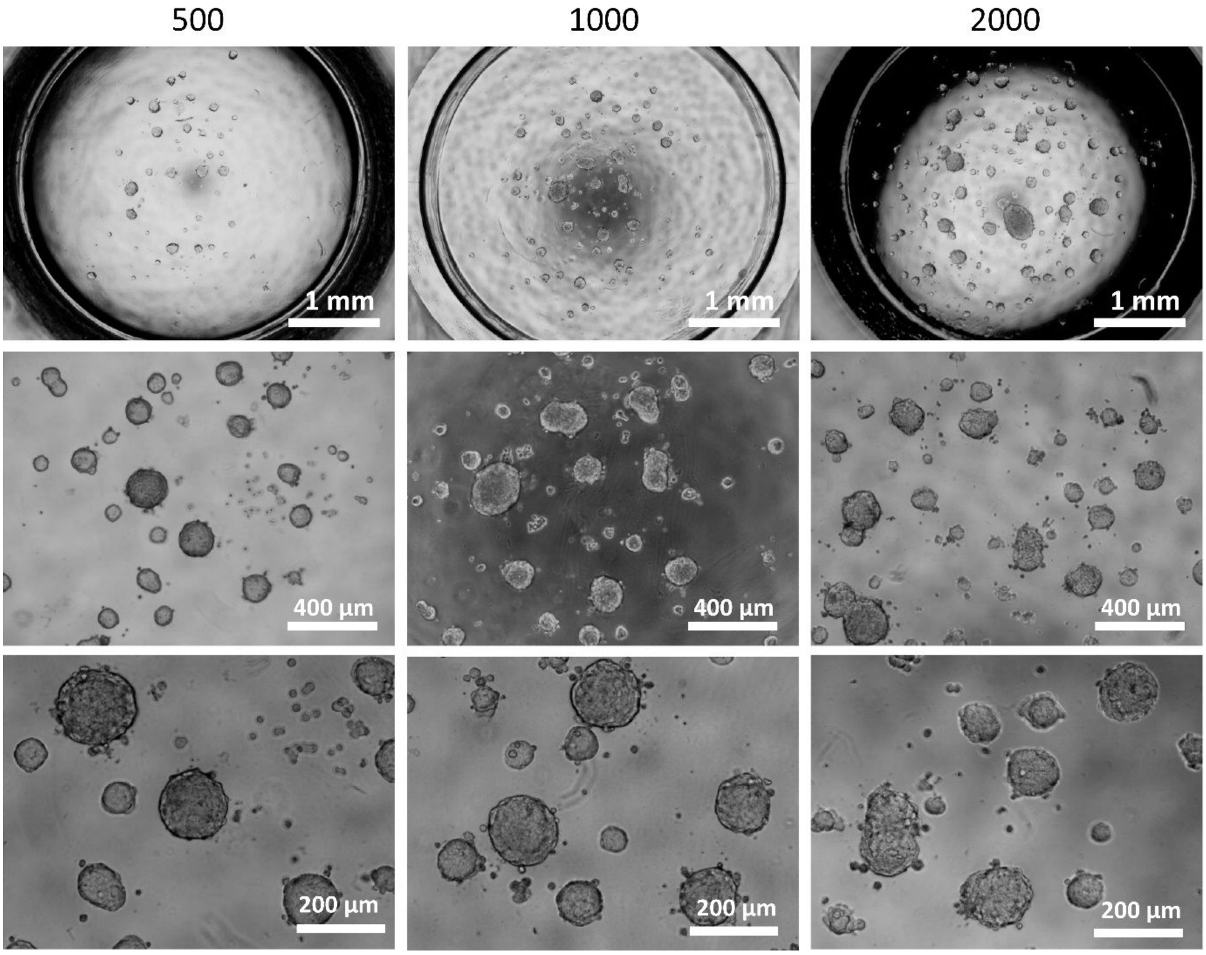

Figure S18. Different cell numbers (500-2000) per well were seeded on 17 mM LBA gel, and cell morphologies were visualized at day 5 by optical contrast microscopy with different magnifications.

No significant difference on spheroid size was observed when seeding different cell numbers. Therefore, 2000 cells per well were used for cell culture experiments, unless stated otherwise.

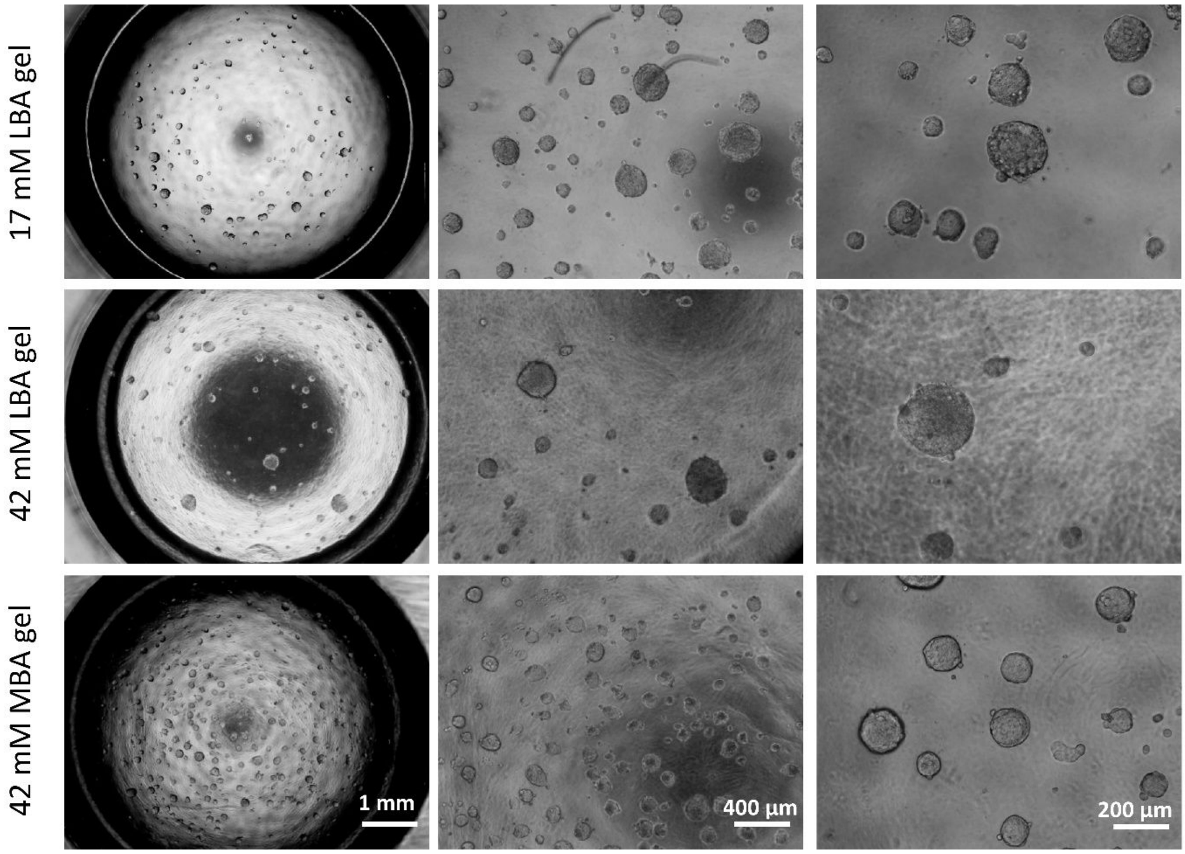

Figure S19. HepG2 cells were grown on LBA amphiphile at 17 mM LBA gel, 42 mM LBA gel, and 42 mM MBA gel, respectively, and cell morphologies were observed at day 3 by optical contrast microscopy with different magnificent. More spheroids were generated on 42 mM MBA amphiphile gel.

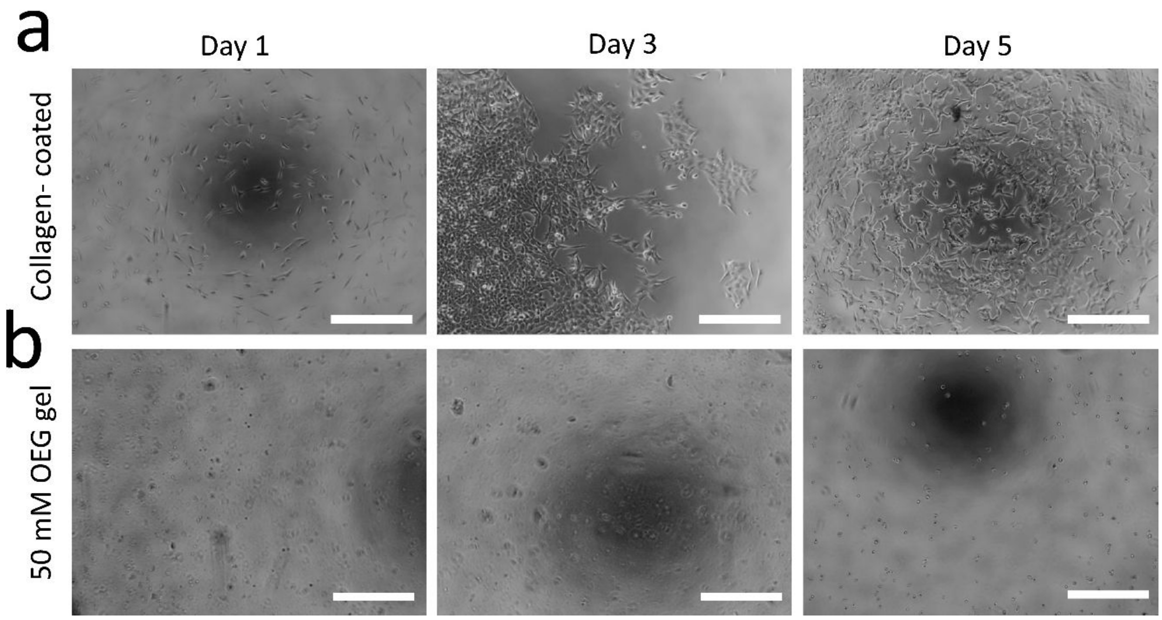

Figure S20. HepG2 cells on plated in collagen-coated well plates and 50 mM OEG hydrogels, respectively at a cell number of 1000 per well. Cells were grown over time by optical contrast microscopic observation. The scale bar is 400 μm.

HepG2 cells exhibited spread-out morphology when cultured on collagen-coated plates, while only few cells survived on the surface of the OEG gel. These results indicate that carbohydrate ligands are essential for promoting formation of HepG2 spheroids.

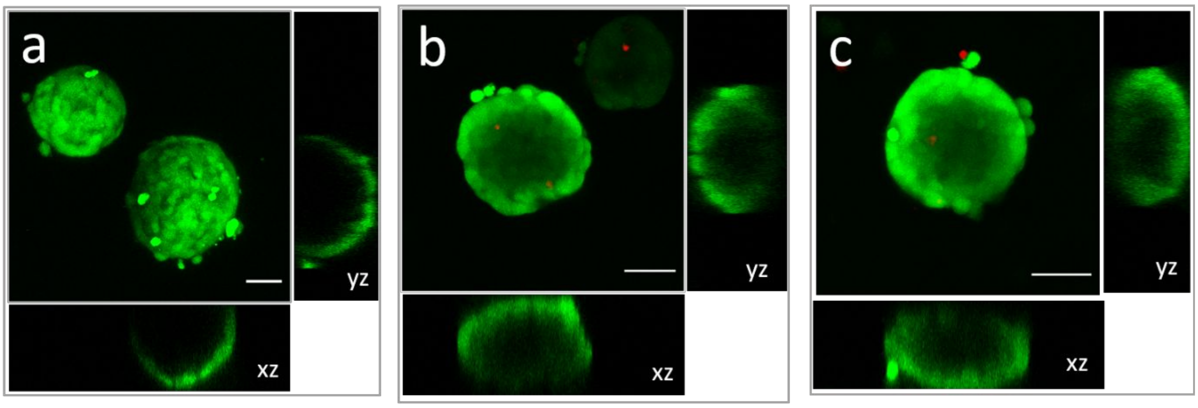

Figure S21. cell viability assay. Living-dead staining of spheroids on 17 mM LBA gel (a), 42 mM LBA gel (c) and 42 mM MBA gel (c) at day 5: living: calcein AM and dead: propidium iodide. The multilayer cells were observed in xz and yz axials from confocal microscopy images. The images were acquired at a certain plane along the focal axis (z). The scale bar is 50 μm.

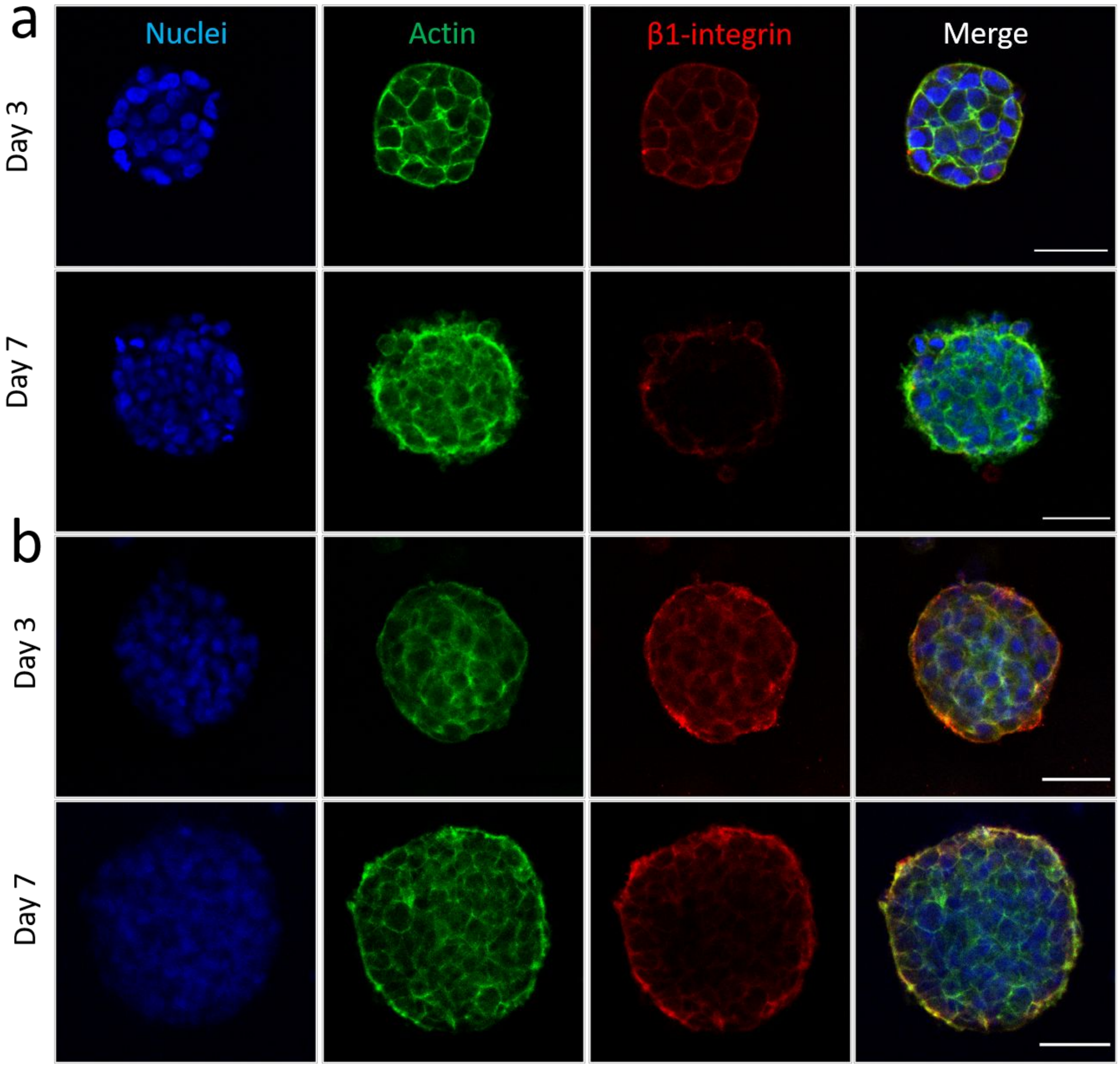

Figure S22. Confocal microscopy of stained spheroids on 17 mM LBA gel (a) and 42 mM MBA gel (b) at day 3 and day 7: Blue: nucleus; green: actin; red: β1-integrin; and merged images. The scale bar is 50 μm.

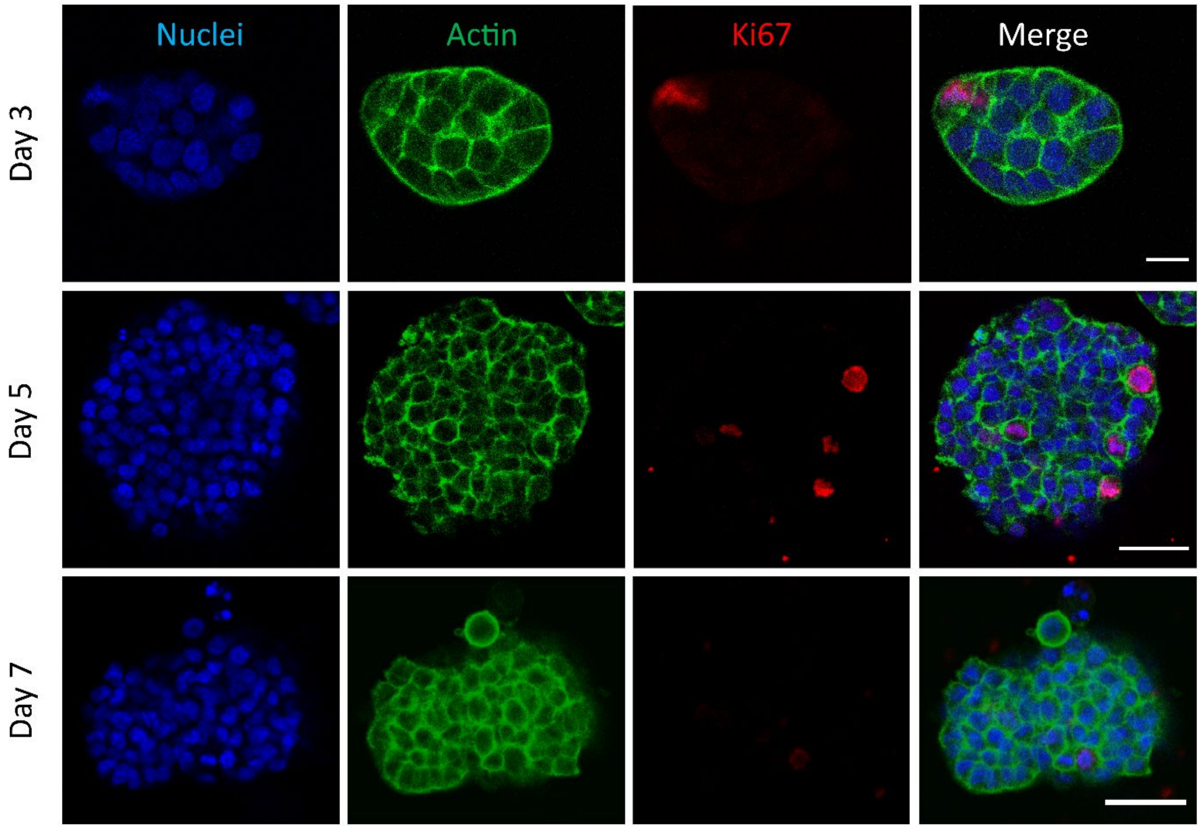

Figure S23. Proliferation assay for spheroid formation on 17 mM LBA gels at day 3, day 5 and day 7 (blue: nuclei; green: actin; red: Ki67; and merged images) by confocal microscopy. The scale bar is 50 μm.

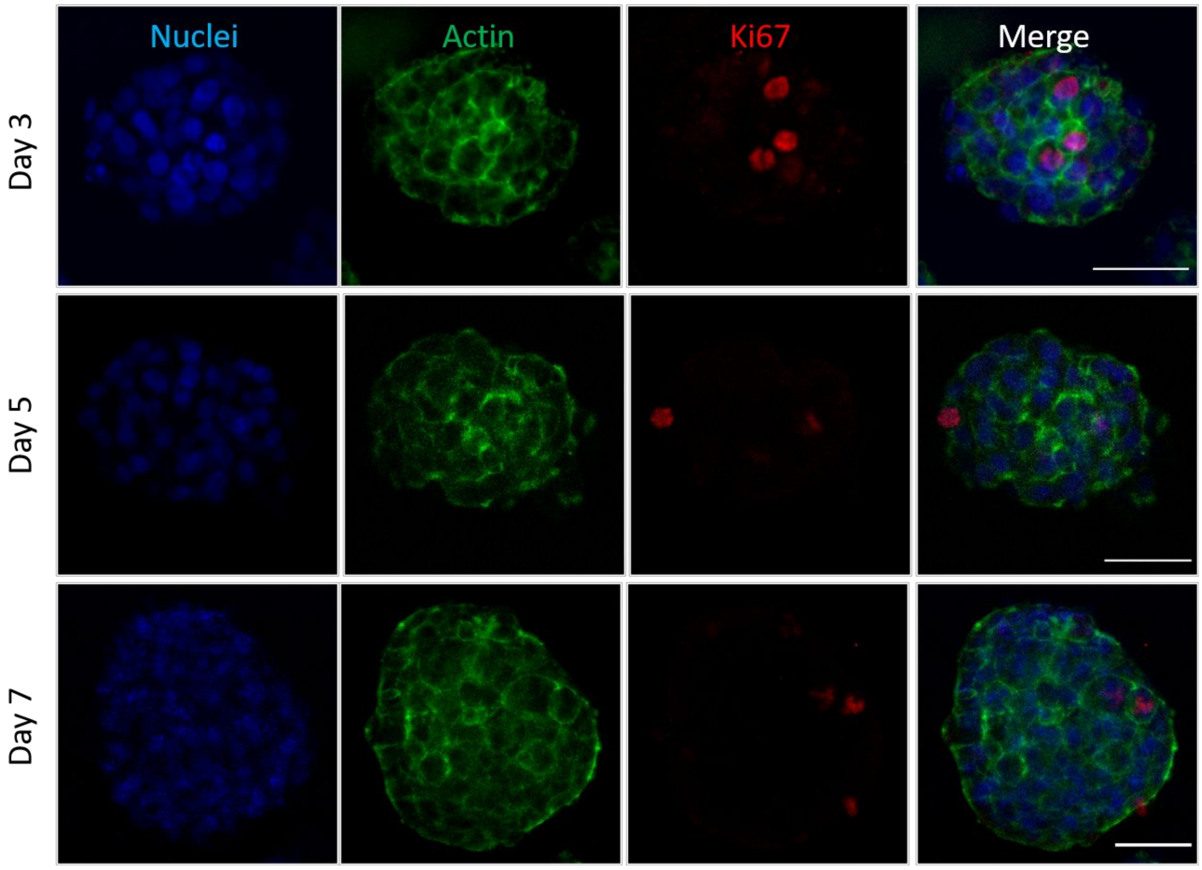

Figure **S24**. Proliferation assay for spheroids formation on 42 mM MBA gels (b) at day 3 and day 7 (blue: nuclei; green: actin; red: Ki67; and merged images) by confocal microscopy observation. The scale bar is 50 μm.

4.7. Cell culture experiments in the presence of Butyl-LBA and Butyl-MBA

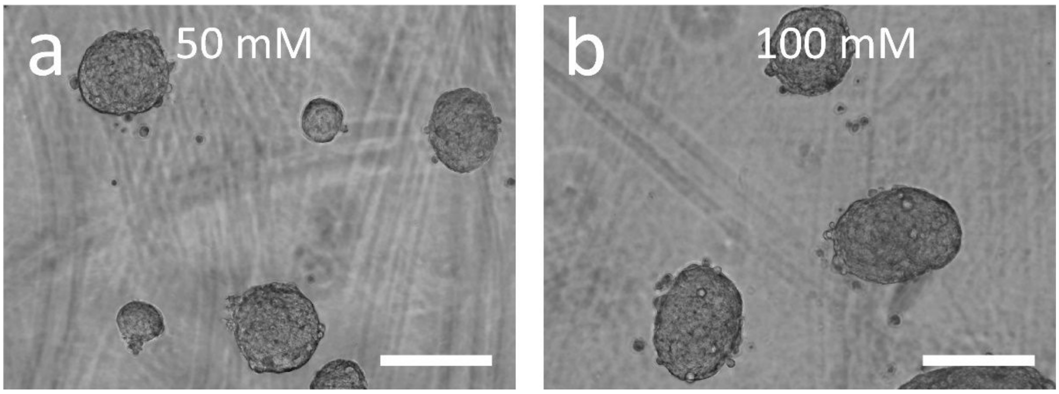

Figure S25. Representative bright-field images of cells treated with 50 mM and 100 mM GalNAc, respectively. The HepG2 cells still forms spheroids with the presence of a low concentration of GalNAc. The scale bar is 200 μm.

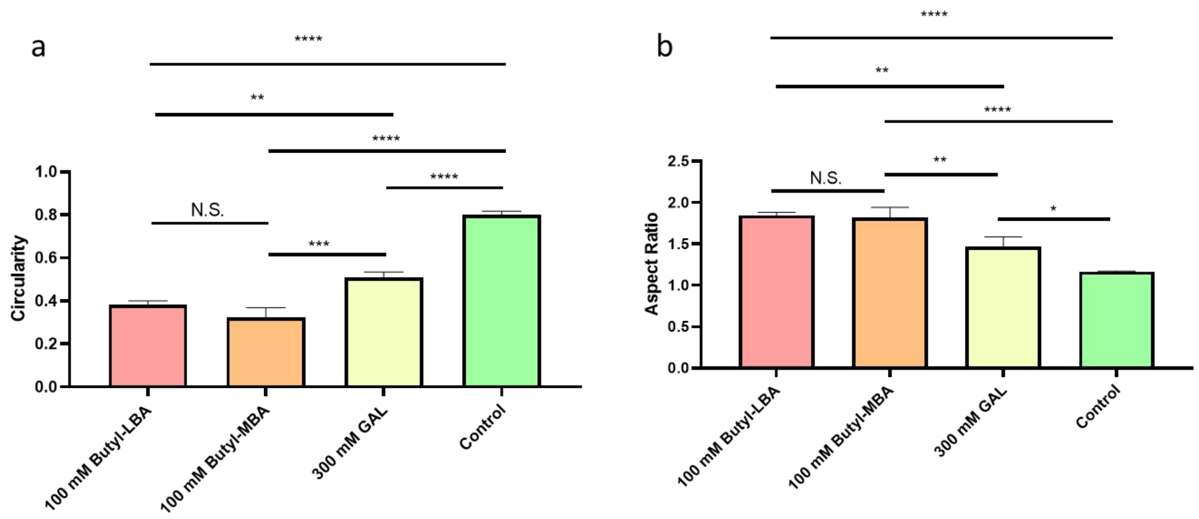

Figure S26. Quantitative analysis of circularity (a) and aspect ratio (b) of cell spheroids or clusters grown on a 17 mM LBA hydrogel. The HepG2 cells were treated with 100 mM Butyl-LBA, 100 mM Butyl-LBA, and 300 mM GalNAc, respectively. Data is presented as mean ± SEM. The statistical significance was determined using unpaired two-sided t-test. P values of statistical significance are represented as * $P < 0.05$, ** $P < 0.01$, *** $P < 0.001$, N.S., not significant. For each group, 10-20 spheroids or clusters were analyzed.

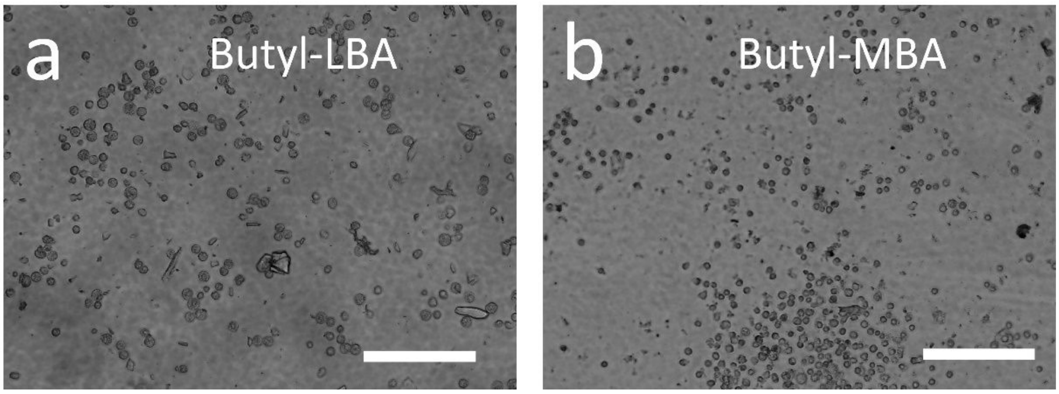

Figure S27. Representative bright-field images of cells treated with 300 mM Butyl-LBA (left) and 300 mM Butyl-MBA, respectively. The scale bar is 200 μm.

References

(1) Williams, E. G. L.; Hutt, O. E.; Hinton, T. M.; Larnaudie, S. C.; Le, T.; MacDonald, J. M.; Gunatillake, P.; Thang, S. H.; Duggan, P. J. Glycosylated Reversible Addition–Fragmentation Chain Transfer Polymers with Varying Polyethylene Glycol Linkers Produce Different Short Interfering RNA Uptake, Gene Silencing, and Toxicity Profiles. *Biomacromolecules* **2017**, *18* (12), 4099–4112. https://doi.org/10.1021/acs.biomac.7b01168.

(2) Fernandez-Castano Romera, M.; Lafleur, R. P. M.; Guibert, C.; Voets, I. K.; Storm, C.; Sijbesma, R. P. Strain Stiffening Hydrogels through Self-Assembly and Covalent Fixation of Semi-Flexible Fibers. *Angew. Chem. Int. Ed.* **2017**, *56* (30), 8771–8775. https://doi.org/10.1002/anie.201704046.

(3) Meazza, L.; Foster, J. A.; Fucke, K.; Metrangolo, P.; Resnati, G.; Steed, J. W. Halogen-Bonding-Triggered Supramolecular Gel Formation. *Nat. Chem.* **2013**, *5* (1), 42–47. https://doi.org/10.1038/nchem.1496.
(4) Cui, J.; Shen, Z.; Wan, X. Study on the Gel to Crystal Transition of a Novel Sugar-Appended Gelator. *Langmuir* **2010**, *26* (1), 97–103. https://doi.org/10.1021/la9021382.
